# Selective and Genetic Constraints on Pneumococcal Serotype Switching

**DOI:** 10.1371/journal.pgen.1005095

**Published:** 2015-03-31

**Authors:** Nicholas J. Croucher, Lisa Kagedan, Claudette M. Thompson, Julian Parkhill, Stephen D. Bentley, Jonathan A. Finkelstein, Marc Lipsitch, William P. Hanage

**Affiliations:** 1 Department of Infectious Disease Epidemiology, Imperial College London, London, United Kingdom; 2 Center for Communicable Disease Dynamics, Department of Epidemiology, Harvard T. H. Chan School of Public Health, Boston, Massachusetts, United States of America; 3 Department of Epidemiology and Department of Immunology and Infectious Diseases, Harvard T. H. Chan School of Public Health, Boston, Massachusetts, United States of America; 4 Pathogen Genomics, The Wellcome Trust Sanger Institute, Wellcome Trust Genome Campus, Hinxton, Cambridge, United Kingdom; 5 Department of Population Medicine, Harvard Medical School and Harvard Pilgrim Health Care Institute, Boston, Massachusetts, United States of America; 6 Division of General Pediatrics, Boston Children’s Hospital, Boston, Massachusetts, United States of America; Uppsala University, Sweden

## Abstract

*Streptococcus pneumoniae* isolates typically express one of over 90 immunologically distinguishable polysaccharide capsules (serotypes), which can be classified into “serogroups” based on cross-reactivity with certain antibodies. Pneumococci can alter their serotype through recombinations affecting the capsule polysaccharide synthesis (*cps*) locus. Twenty such “serotype switching” events were fully characterised using a collection of 616 whole genome sequences from systematic surveys of pneumococcal carriage. Eleven of these were within-serogroup switches, representing a highly significant (*p* < 0.0001) enrichment based on the observed serotype distribution. Whereas the recombinations resulting in between-serogroup switches all spanned the entire *cps* locus, some of those that caused within-serogroup switches did not. However, higher rates of within-serogroup switching could not be fully explained by either more frequent, shorter recombinations, nor by genetic linkage to genes involved in β–lactam resistance. This suggested the observed pattern was a consequence of selection for preserving serogroup. Phenotyping of strains constructed to express different serotypes in common genetic backgrounds was used to test whether genotypes were physiologically adapted to particular serogroups. These data were consistent with epistatic interactions between the *cps* locus and the rest of the genome that were specific to serotype, but not serogroup, meaning they were unlikely to account for the observed distribution of capsule types. Exclusion of these genetic and physiological hypotheses suggested future work should focus on alternative mechanisms, such as host immunity spanning multiple serotypes within the same serogroup, which might explain the observed pattern.

## Author Summary

*Streptococcus pneumoniae* is a major respiratory pathogen responsible for a high burden of morbidity and mortality worldwide. Current anti-pneumococcal vaccines target the bacterium’s polysaccharide capsule, of which at least 95 different variants (‘serotypes’) are known, which are classified into ‘serogroups’. Bacteria can change their serotype through genetic recombination, termed ‘switching’, which can allow strains to evade vaccine-induced immunity. By combining epidemiological data with whole genome sequencing, this work finds a robust and unexpected pattern of serotype switching in a sample of bacteria collected following the introduction of routine anti-pneumococcal vaccination: switching was much more likely to exchange one serotype for another within the same serogroup than expected by chance. Several hypotheses are presented and tested to explain this pattern, including limitations of genetic recombination, interactions between the genes that determine serotype and the rest of the genome, and the constraints imposed by bacterial metabolism. This provides novel information on the evolution of *S*. *pneumoniae*, particularly regarding how the bacterium might diversify as newer vaccines are introduced.

## Introduction

*Streptococcus pneumoniae* is a human nasopharyngeal commensal bacterium and important respiratory pathogen. The ability of the pneumococcus to cause invasive disease appears critically dependent upon its polysaccharide capsule [1], of which at least 95 immunologically-distinguishable capsular variants (serotypes) are known [2–7]. This structure inhibits the recognition of subcapsular protein antigens by the adaptive immune system and the binding of phosphorylcholine residues by C-reactive protein, thereby reducing the rate of complement deposition on the bacterial surface [8]. Children develop anticapsular antibodies after exposure to the bacterium, although evidence for their impact on disease risk is mixed [9]. Data indicating this immune response provides protection against nasopharyngeal carriage has been found for only a few serotypes [10–12], though mathematical models suggest subtle effects on carriage may exist and help maintain the high level diversity of serotypes observed in pneumococcal populations [13].

In contrast to natural immunity, that induced by the seven-valent protein conjugate polysaccharide vaccine (PCV7) reduces acquisition of vaccine serotypes in the nasopharynx by 50% [14] or more [15]. Following the introduction of PCV7, a substantial fall in the carriage of the seven vaccine serotypes (4, 6B, 9V, 14, 18C, 19F and 23F) was observed without any substantial overall reduction in the rates of pneumococcal colonisation [16,17]. This was primarily the result of an increase in the rate of carriage of non-vaccine type strains, termed ‘serotype replacement’ [18–20]. In some cases non-vaccine type strains are closely related to vaccine type strains from which they have been derived by ‘serotype switching’ [21]. In these cases, strains have altered their serotype through the acquisition of a different capsular polysaccharide synthesis (*cps*) locus via genetic transformation.

Many serotypes, though distinguishable by certain antisera (called "factor sera"), nonetheless may be clustered into “serogroups” based on cross-reactivity with other antisera; these groups have often been found to correspond to sets of similar polysaccharide structures [2]. It was originally hoped that PCV7 would provide cross-protection against ‘vaccine-related’ serotypes: those within the same serogroups as a serotype included in the vaccine [22]. However, the only case in which such an effect was observed was the protection against colonisation with serotype 6A resulting from the inclusion of serotype 6B in PCV7; several other vaccine-related types increased in prevalence post-PCV7 [23]. Contributing to this pattern was an apparent tendency for serotype switches to exchange one serotype for another within the same serogroup more often than expected by chance: of nine switches inferred using multilocus sequence typing (MLST) in a systematic collection of carried pneumococci from Massachusetts, three were within-serogroup (*p* = 0.043) [24]. Hence genotypes successful prior to the introduction of PCV7 were able to persist expressing a similar capsule that was not recognised by vaccine-induced immunity. This was not a pattern expected *a priori*, as random acquisition of a new serotype from the full, diverse set of non-vaccine type capsules should usually result in a change of serogroup.

This work assesses the relative likelihood of alternative explanations of the observed pattern of serotype switching based on whole genome sequencing data from this Massachusetts-based collection [25]. The first is that the detected propensity for within-serogroup serotype switching is a consequence of the constraints of genetic transformation. Pneumococcal *cps* loci are typically 10–30 kb in size, usually necessitating a similarly long recombination to cause a change of serotype [26], whereas homologous recombinations have an exponential distribution of sizes with a mean length of between two and seven kilobases [27–29]. Aside from known exceptions such as serogroups 7, 17, 33 and 35, the *cps* loci corresponding to a single serogroup are closely related [2]. Therefore, within-serogroup serotype switching may be accomplished through relatively short, more frequent, recombination events that do not replace the entire *cps* locus [30]. Another possibility is that patterns of serotype switching may be affected by the flanking *pbp1a* and *pbp2x* genes, which are crucial in determining β–lactam resistance. As resistance is associated with a limited number of serotypes [31], and long recombinations that change serogroup could lead to the acquisition of resistance [32] or risk reversing any beneficial acquisition of resistance-associated *pbp1a* or *pbp2x* alleles [29,33], it may be that maintenance of a strain’s β–lactam susceptibility (or lack thereof) affects the patterns of serotype switching.

Alternatively, rather than representing a limitation on the rate of recombination, the pattern of switching may reflect the consequence of constraints imposed by epistatic interactions with other loci in the chromosome [26]. Such limitations may reflect physiological or metabolic specialisation to production of particular capsule types; alternatively, it may be important to co-ordinate the expression of particular serogroups with certain alleles of immunogenic surface proteins, in order to maintain discordant sets of antigens in distinct lineages [34]. The last explanation to be considered, which also involves the host immune response, relies on the presence of natural or vaccine-induced antibodies that cross-react with all serotypes within a serogroup. This would lead to elevated rates of co-colonisation between strains of the same serogroup, as bacteria would be confined to a subpopulation of hosts that do not exhibit immunity to either of their capsules. A consequence of this would be increased genetic exchange between members of the same lineage, including an elevated rate of within-serogroup serotype switching.

## Results

### Association between serotypes and genotypes

**Fig 1:**
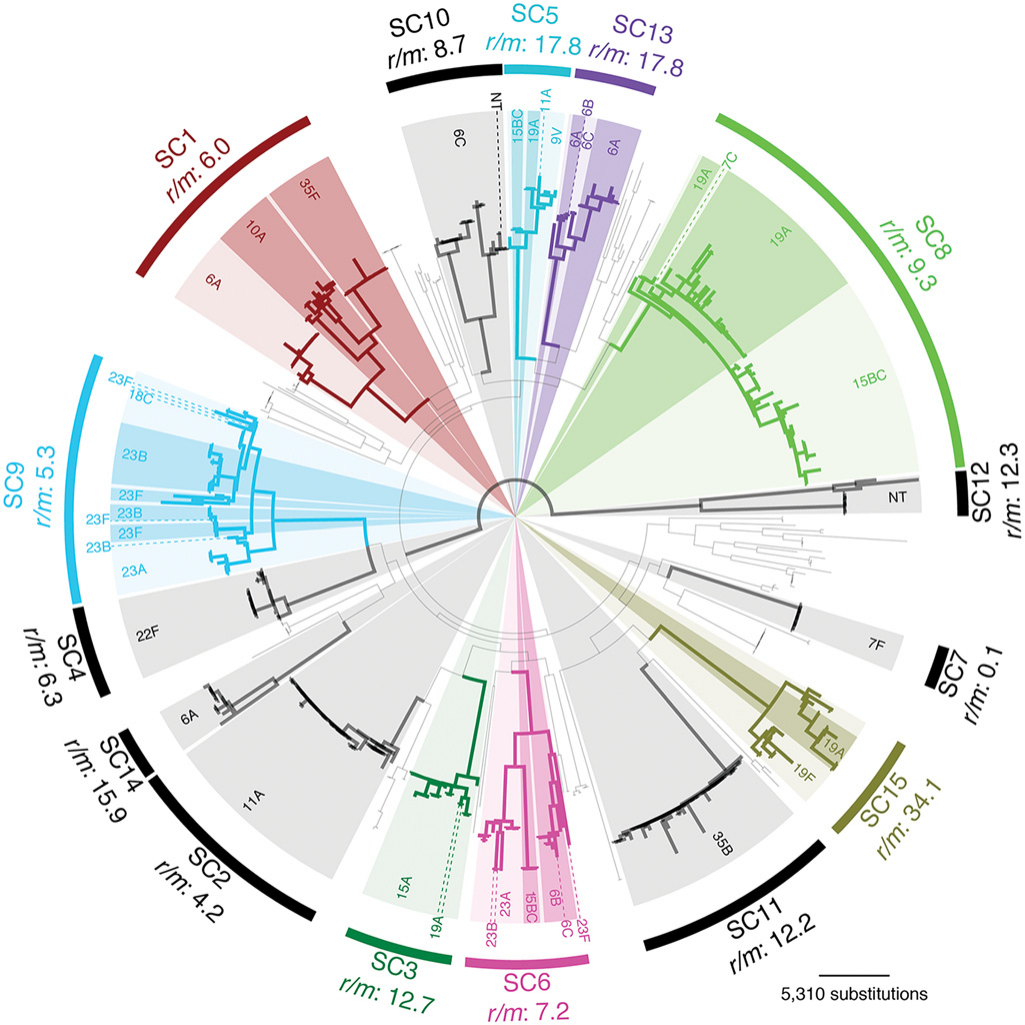
Prevalence of serotype switching in the overall population. The maximum likelihood phylogeny derived from a codon alignment of ‘core’ protein coding sequences is displayed, with fifteen monophyletic sequence clusters (SC1-SC15) labelled by arcs around the edge of the tree. Those sequence clusters in which no serotype switching was observed are marked by black arcs. Those sequence clusters in which multiple serotypes were observed are individually coloured, with the most prevalent serotype represented by light shading, and alternative serotypes indicated by darker shading or dashed lines.

Of the 616 draft genomes previously analysed [25], 491 fell into fifteen monophyletic sequence clusters (SCs) of related isolates within which serotype switching could be investigated (Fig. 1). These sequence clusters included representatives of 19 serotypes spread across eleven serogroups. In seven sequence clusters, the serotype was stable across the clade, leaving eight clusters in which at least one switch had occurred.

**Fig 2:**
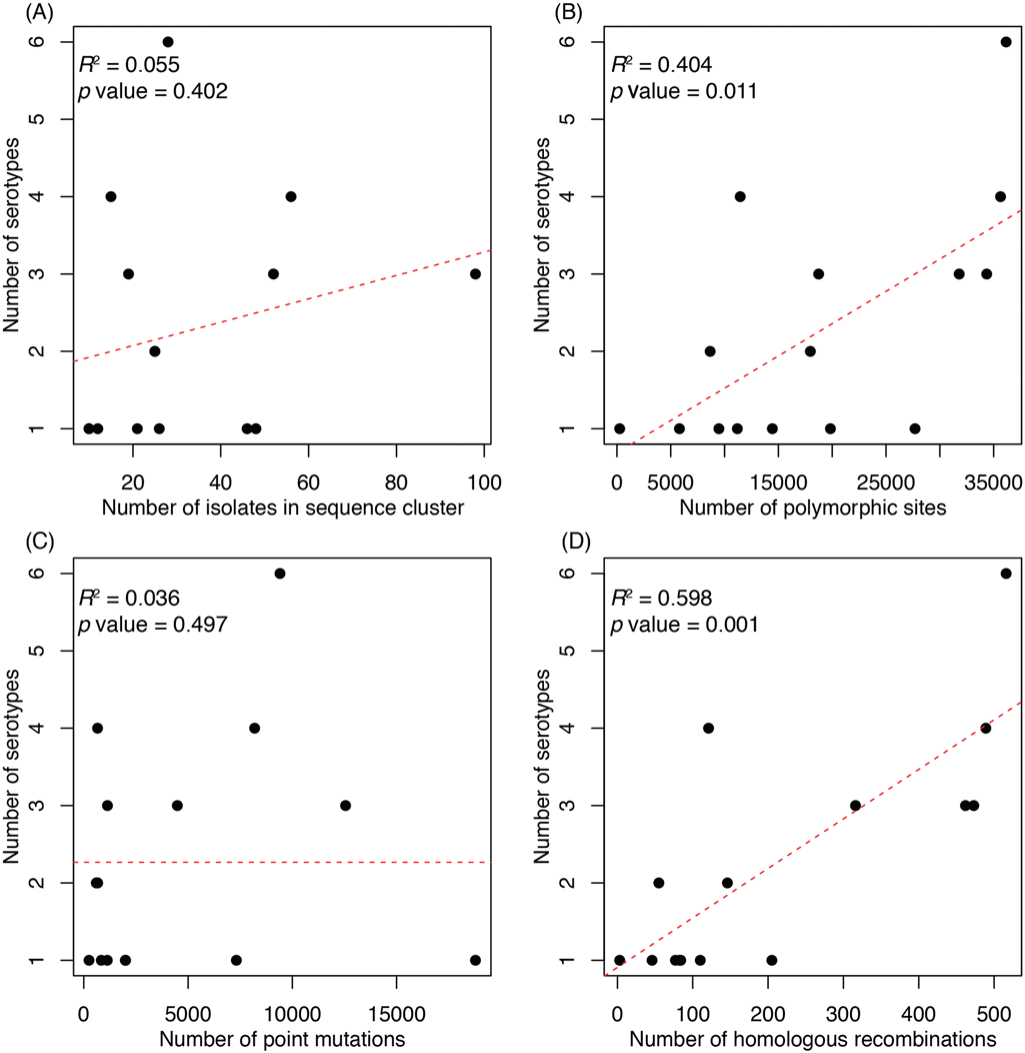
Associations between properties of a sequence cluster and its capsular diversity. For each of the fifteen monophyletic sequence clusters, the number of serotypes it contained was plotted against (A) the number of representatives of the sequence cluster; (B) the overall genetic diversity of the sequence cluster, as represented by the number of polymorphic sites identified from whole genome alignments; (C) the number of point mutations reconstructed as having occurred over the history of the sequence cluster; and (D) the number of homologous recombination events reconstructed as having occurred over the history of the sequence cluster.

There was no positive correlation between the number of isolates sampled within a sequence cluster and the number of serotypes it contained (Fig. 2A). However, a significant correlation was observed between the number of polymorphic sites, as ascertained through a lineage-specific analysis of whole genome alignments, and the number of serotypes in a cluster (Fig. 2B). When these sites were split into the number of point mutations and homologous recombination events per sequence cluster (see Methods), there was no significant relationship with the former, whereas the latter had the strongest correlation of any measure of genetic diversity (Fig. 2C and D). Therefore sequence clusters that exhibit serotype diversity are those that have experienced more recombination events throughout their genome over their observable evolutionary history, either as a consequence of a high rate of recombination over their recent diversification, or lower recombination rates over a longer period of time.

### Frequent within-serogroup switching

Across the species, the 95 currently known *S*. *pneumoniae* serotypes are divided into 48 serogroups such that 2.2% of comparisons between different serotypes are serogroup concordant. However, many serotypes are rarely observed; the 32 serotypes observed across all 616 isolates from Massachusetts were distributed such that 3.0% of serotype comparisons were serogroup concordant. Seventeen serotypes were observed within the eight sequence clusters that appear to contain examples of serotype switching; 7.2% of all pairwise comparisons between these serotypes were serogroup-concordant, suggesting these isolates expressed a comparatively limited number of serogroups. Yet when only considering the subset of these comparisons where the different serotypes were found within the same sequence cluster, 29% were serogroup concordant (S1 Table), as some individual sequence clusters were associated with multiple serotypes from the same serogroup. The significance of this enrichment was assessed by a permutation test that randomly assigned serotypes to these eight sequence clusters according to the number of serotypes originally observed in the sequence cluster. This found the high level of serogroup-concordant comparisons within sequence clusters to be statistically significant (*p* = 0.0007 from 10,000 permutations). As sequence clusters were identified through a clustering algorithm [25], this observation is independent of any phylogenetic analyses. Therefore this test indicates isolates with similar core genomes are more likely to express capsules of the same serogroup than expected from the overall capsular diversity of the isolate collection, even when accounting for isolates sharing the same serotype through common descent.

**Fig 3:**
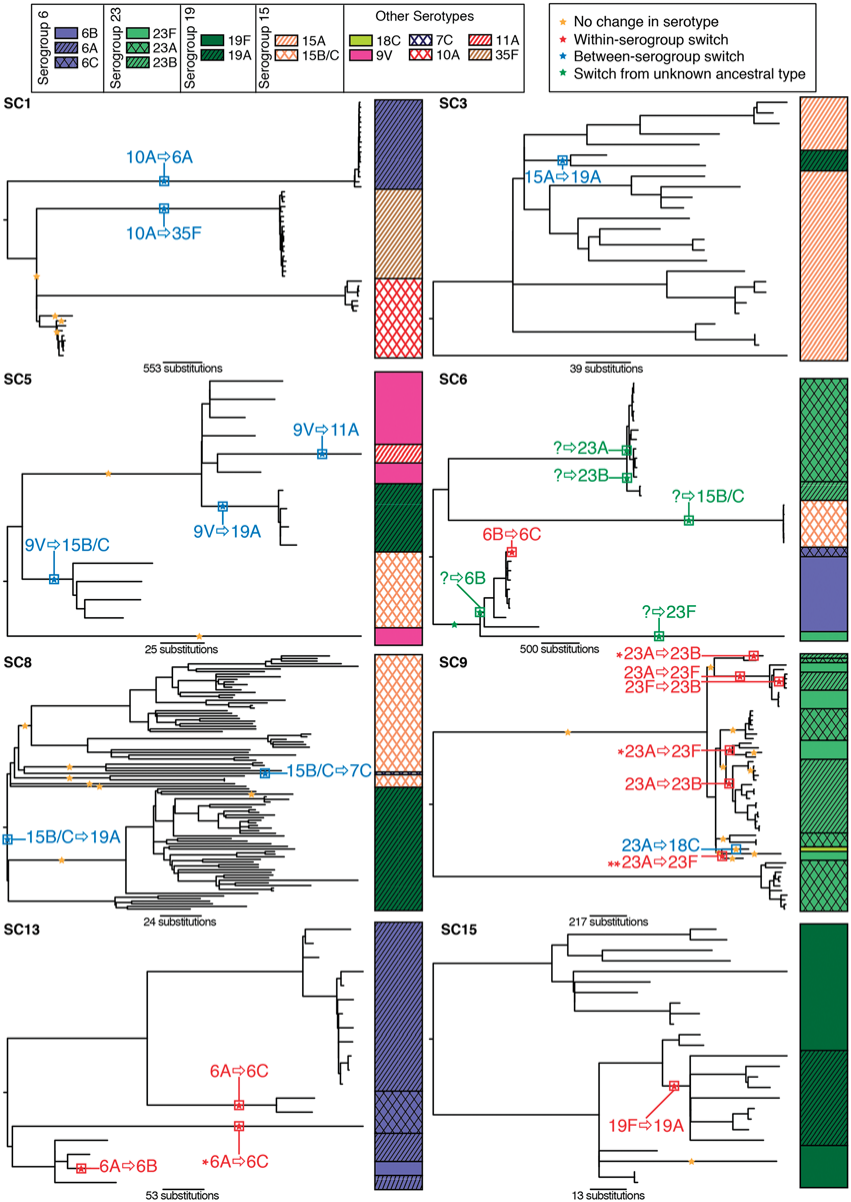
Serotype switching events within sequence clusters. The evolution of each of the monophyletic sequence clusters highlighted in Fig. 1 was reconstructed from a whole genome alignment (see Methods). The resulting phylogenies are annotated with the serotype of the isolates. Stars on the branches of each phylogeny mark instances where putative recombinations affected the *cps* locus. These stars are coloured according to the inferred consequences of the recombination. Where these sequence imports led to a change in serotype, this is annotated: in red for within-serogroup switches, in blue for between-serogroup switches, and in green, where the ancestral serotype cannot be reliably determined.

Greater resolution can be achieved by using the phylogenetic analysis of the whole genome alignments for each of the sequence clusters in which serotype switching occurred (S1 Text & Fig. 3) [25]. These attempt to reconstruct the history of the lineages more accurately by identifying recombination events and excluding the horizontally acquired polymorphisms they introduce from the point mutations used to generate the tree [29]. These reconstructions found that some within-serogroup switching occurred multiple times in parallel in the same sequence cluster: for instance, in SC9 the ancestral type of 23A was replaced by 23B on three occasions, and 23F on three occasions. Similarly within SC13, the ancestral 6A type is exchanged for 6C on two occasions in parallel. In both cases, these examples of convergent evolution are independently supported by the divergence between the *cps* loci, encoding the same serotype, imported by separate capsule switching events (S1 Fig). A further switch to 6C, this time from 6B, was observed within SC6. However, the other changes within this sequence cluster cannot be reliably inferred as they occur on long branches that are difficult to reconstruct. These ‘missing data’ include at least two between-serogroup switches, but also likely within-serogroup switches given that the cluster includes 23A, 23B and 23F isolates.

By contrast, each of the robustly inferred between-serogroup switches occurred only once. Hence this more detailed analysis revealed an even greater tendency to swap a serotype for one within the same serogroup than the previous results. Overall, eleven of the twenty serotype switches (55%) fully defined in this population did not result in a change in serogroup. A further permutation test, in which the derived serotypes were randomly assigned to each ancestral serotype through resampling without replacement 10,000 times (see Methods), found this result to be highly significant (*p* < 0.0001). Therefore this collection provides robust evidence that within-serogroup switching is more common than expected by chance, relative to the rate of between-serogroup switching.

### Length of serotype switching recombinations

The first hypothesis that might explain this observation concerns the mechanics of recombination: the smaller recombinations necessary to alter a *cps* locus to a related sequence generating a similar serotype may be more frequent than the replacement of the entire locus. The within-serogroup switches observed in this collection all require only minor changes in the *cps* locus: the serogroup 23 *cps* loci are thought only to differ in the sequence of their *wzy* polymerase, as is also the case for serotypes 19A and 19F [35]. Similarly, switching between serotypes 6A and 6B can occur through a single polymorphism in the *wciP* gene [30], while 6A and 6C only differ in polymorphisms in their *wciN* gene [35].

**Fig 4:**
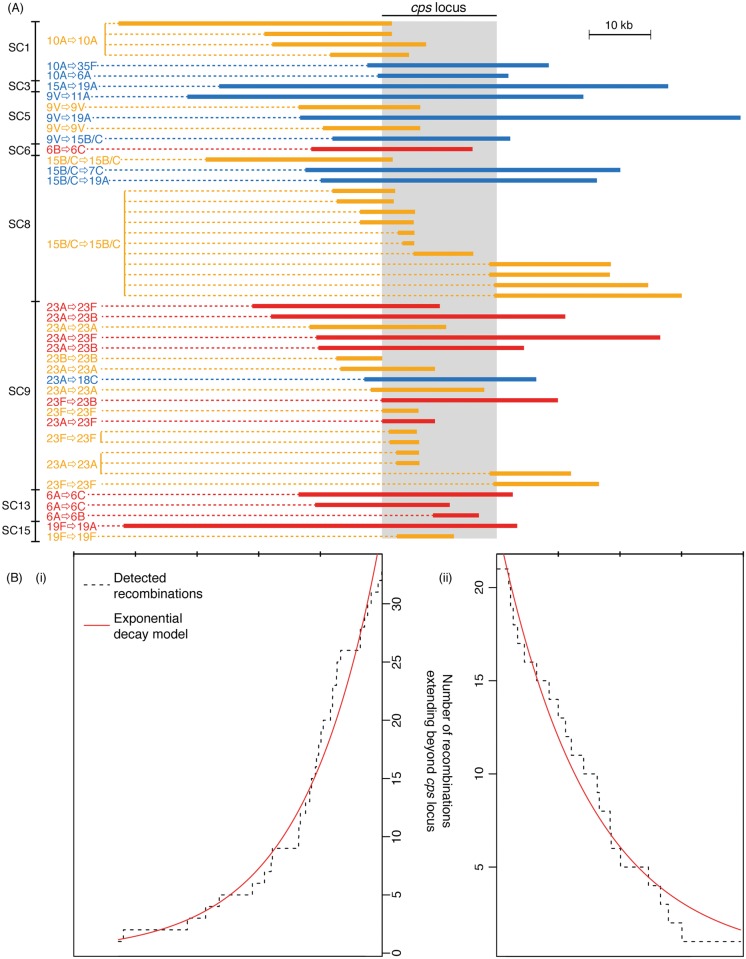
Extent of recombinations affecting the *cps* locus. (A) Positions of recombinations affecting the *cps* locus. Each of the recombinations identified in Fig. 3 is represented by a horizontal bar coloured according to the inferred impact of the recombination. Recombinations causing a change in serotype are uniquely annotated to correspond with Fig. 3. The grey column represents the extent of the *cps* locus; the width displayed is that of the longest *cps* locus across the different sequence clusters, and each recombination is scaled relative to the positions of this *cps* locus’ boundaries. (B) Extent of recombinations affecting the *cps* locus. The degree to which the recombinations displayed in (A) impinge on the flanking regions is summarised, with the dashed line representing the falling numbers of recombinations extending to bases further removed from the *cps* locus edges (displayed on the same scale as (A)). The red lines represent the fit of exponential decay curves to these trends. The tick marks above the plots represent 10 kb intervals.

**Fig 5:**
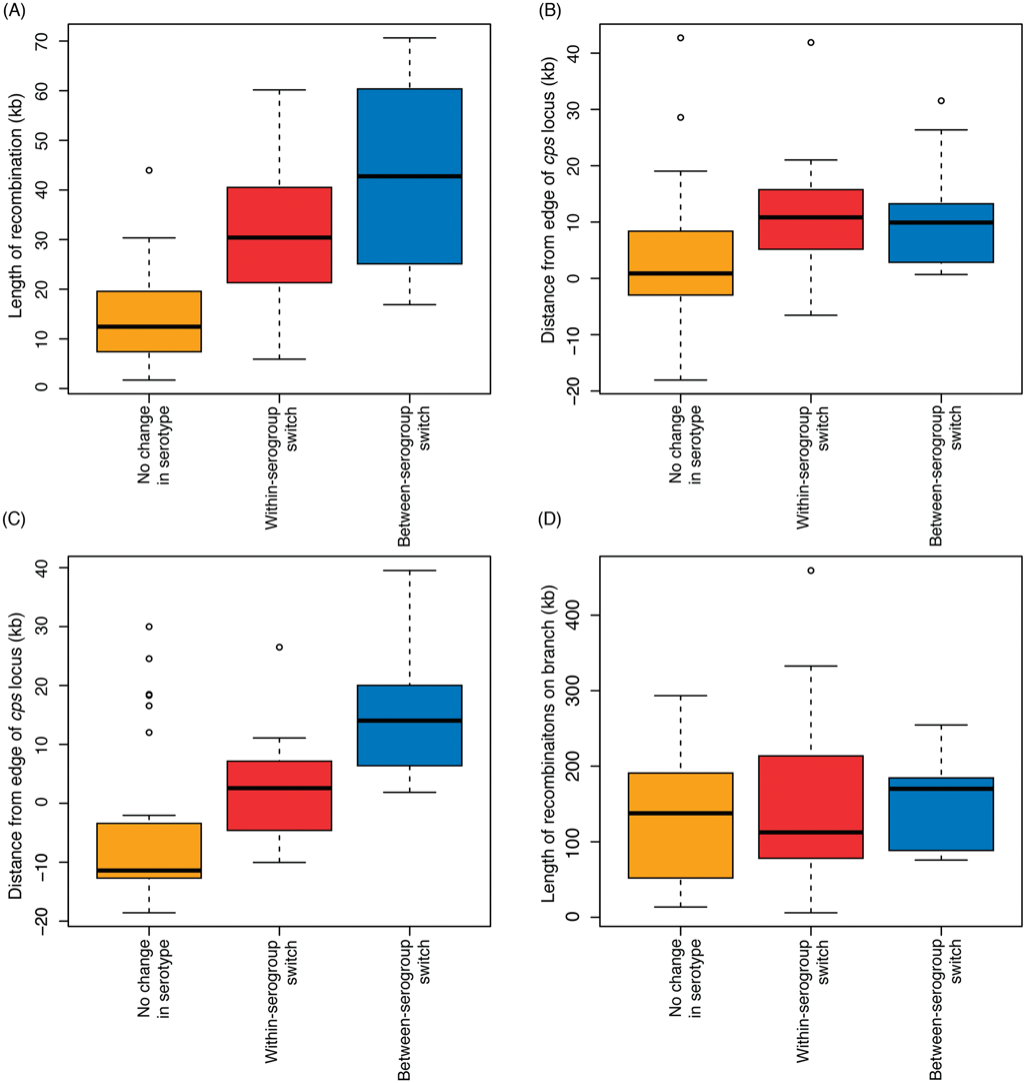
Properties of recombinations affecting the *cps* locus. In each boxplot, the recombinations affecting the *cps* locus are grouped according to their inferred impact on serotype. The first three graphs describe the recombinations affecting the *cps* locus. The values represent (A) overall length of recombination, (B) position of the 5’ edge of the recombination relative to the 5’ edge of the *cps* locus, and (C) position of the 3’ edge of the recombination relative to the 3’ edge of the *cps* locus. In the latter two plots, negative values indicate the recombination boundary is within the *cps* locus. The boxplot in (D) represents the total length of genome-wide recombinations on the same phylogenetic branch as the serotype switching events.

Including only the switch from serotype 6B to 6C from SC6 (see S1 Text), the analysis of whole genome alignments used to generate Fig. 3 identified 50 putative homologous recombination events that overlapped the *cps* loci of the relevant reference genome sequences (Fig. 4). The nine recombinations that resulted in a change in serogroup had a median length of 42.7 kb, and all spanned the entire *cps* locus. The eleven recombinations that led to a within-serogroup switch had a shorter median length, at 30.4 kb (Fig. 5A). These almost all spanned the 5’ region of the *cps* locus, but in some cases did not extend so far as the 3’ end. In two cases, this could be ascribed to the presence of the *rml* rhamnose synthesis operon at the 3’ end of the *cps* locus. Both the acquisition of the 6B capsule in SC13, and the 6C capsule in SC6, terminated within this gene cluster; however, the *rml* operon is found in several serogroups [2], and recombinations causing between-serogroup switches have previously been observed to end within it [29]. Only three recombination events were observed where the 3’ recombination breakpoint occurred in a region that might be considered serogroup-specific. Two of these were switches from 23A to 23F within SC9 (Fig. 4). In both cases, the *wzy* polymerase gene that distinguished these *cps* loci was replaced; however, the recombinations were far more extensive than this minimal alteration, as they extended to or beyond the 5’ boundary of the *cps* locus. The switch to 6C within SC13 that ended before the 3’ boundary of the locus was also far more extensive than simply encompassing the *wciN* gene. The overall difference in the distribution of lengths between recombinations causing within- and between-serogroup switches was not significant (Fig. 5; Wilcoxon rank sum test, W = 35, *p* value = 0.29). By contrast, the 30 recombinations inferred to overlap with the *cps* loci that did not affect serotype had a median length of 11.7 kb and were significantly shorter than both the recombinations that alter serogroup (Wilcoxon rank sum test, W = 22, *p* value = 3.4x10^-5^) and those that caused within-serogroup switching (Wilcoxon rank sum test, W = 61, *p* value = 0.0016). As the majority of within-serogroup switches were caused by recombinations long enough to cause changes in serogroup, restrictions on transformation event length cannot fully explain the observed pattern of switching.

There was extensive variation in the boundaries of the three classes of recombination event. For both 5’ and 3’ breakpoints outside the *cps* locus, the distance between the breakpoint and the *cps* locus edge (Fig. 4B) followed an approximately exponential distribution, as previously observed for experimental transformants at the *cps* locus [28]. However, these observed recombinations were longer than the length of transformation events observed experimentally, with a rate of decay approximately an order of magnitude lower in this work: the rate constant for the decline on the left side was 8.07x10^-5^ bp^-1^ (95% confidence interval: 8.06x10^-5^–8.09x10^-5^ bp^-1^), while on the right side it was 6.75x10^-5^ bp^-1^ (95% confidence interval: 6.74x10^-5^–6.77x10^-5^ bp^-1^), as opposed to the previous estimates of ~3.4x10^-4^ bp^-1^. This is likely partly a consequence of the events described in Fig. 4 being composed long “mosaics” of recombinant DNA segments [28], and also potentially representing larger “macrorecombination” events [36].

The *pbp2x* and *pbp1a* genes, crucial determinants of β–lactam resistance, are found 9–10 kb upstream and downstream of the *cps* locus respectively. Seven of the recombinations shown in Fig. 4 affected both, all of which caused a change of serotype. However, they were split between within- and between-serogroup switches, and none significantly affected the strain’s β–lactam resistance (S2 Table & S2 Text). Hence serotype switches did not appear to be driven by selection for the acquisition of β–lactam resistance. Nevertheless, it is possible that maintenance of a genotype’s β–lactam resistance level may counterselect against recombination events that substantially alter *pbp1a* or *pbp2x* [33].

### Adaptation between serotype and genotype

If the distribution of capsular variation does not reflect the properties of genetic transformation, then selection may be important in driving the observed pattern. Possible selective pressures include adaptive immune responses in the host population [34], or physiological constraints relating to the bacterium. To test the latter possibility, capsule-switched variants were constructed in common genetic backgrounds and characterised through recording growth curves.

**Fig 6:**
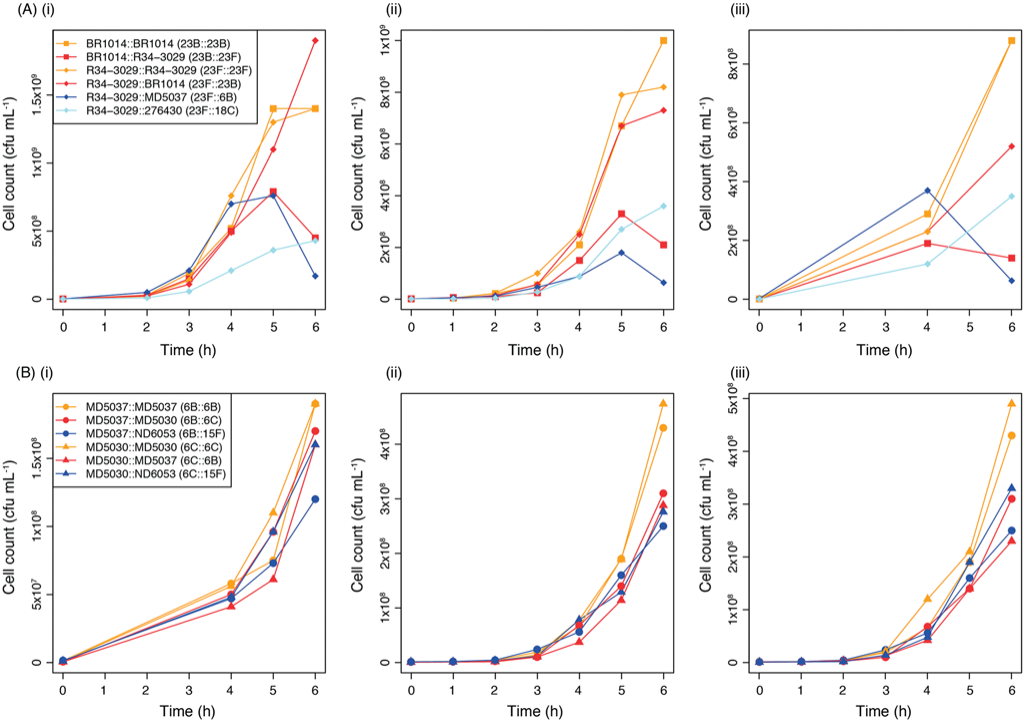
Growth curves comparing the fitness of genetic backgrounds expressing different serotypes. (A) Effect of capsule switching on the growth of two isolates from SC9. Three replicate experiments (i-iii) are shown in which six mutants were compared. Four were constructed based on isolate R34-3029 (diamond points): one in which the native 23F capsule was restored (orange); a within-serogroup switch to the 23B capsule (red); a between-serogroup switch to the 18C capsule (light blue); and a further between-serogroup switch to the 6B capsule (blue). Two other mutants were constructed based on isolate BR1014 (square points): one in which the native 23B capsule was restored (orange), and a within-serogroup switch to 23F (red). (B) Effect of capsule switching on the growth of two isolates from SC13. Three replicate experiments (i-iii) are shown in which six mutants were compared. Three mutants were based on isolate MD5037 (circular points): a restoration of the native 6B capsule (orange); a within-serogroup switch to the 6C capsule (red); and a between-serogroup switch to the 15F capsule (blue). The other three mutants were based on isolate MD5030 (triangular points): a restoration of the native 6C capsule (orange), a within-serogroup switch to the 6B capsule (red); and a between-serogroup switch to the 15F capsule (blue).

The association of SC9 with serogroup 23 was investigated by knocking out the native *cps* locus of isolate R34-3029, and using this genetic background to construct one mutant in which the native 23F capsule type was restored, a second through a within-serogroup switch to capsule type 23B, and two further mutants through between-serogroup switches to 18C and 6B. Capsule type 18C was selected as it was the only non-serogroup 23 capsule type observed within SC9, whereas 6B was not found within SC9 but has similar properties to 23F [37]. Both serogroup 23 mutants were found to grow similarly quickly (Fig. 6A) and substantially faster than the two mutants bearing capsules of other serogroups. This suggested that these bacteria may be adapted to serogroup 23 capsules. However, comparisons of the original isolates used as the sources of the *cps* loci (S2 Fig) indicated that the apparent reduction in fitness of SC9 mutants expressing non-serogroup 23 capsule types might reflect an intrinsically slower growth phenotype associated with these capsule types. Furthermore, when the reciprocal exchange of *cps* loci between isolates BR1014 and R34-3029 was performed, BR1014 grew faster following restoration of its native 23B capsule relative to the variant into which the 23F capsule had been introduced (Fig. 6A). Therefore changes in capsule type were generally associated with a greater reduction in growth rate than reinstatement of the original capsule type; however, this inhibition could be observed regardless of whether the new serotype was of a different serogroup or not.

To test this hypothesis further, six additional mutants were constructed using two SC13 isolates in which the *cps* locus had been knocked out: MD5037, originally of 6B, and MD5030, originally of 6C (Fig. 6B). The 6B, 6C and 15F capsule types were introduced into both. This comparison allowed a more precise test for interactions between the *cps* locus and the rest of the genome than the experiments with SC9 isolates, as SC13 isolates exhibited more consistent growth patterns, and the selected donor of the 15F capsule had a similar growth curve to the recipient isolates (S2 Fig). Consistent with the observations of SC9, the mutants with the restored native capsule grew fastest. However, both the within-serogroup and between-serogroup switched variants exhibited similarly inhibited growth rates. Hence all three capsule types replicated optimally in their native backgrounds, and more slowly when introduced into a non-native background. Therefore the only epistasis between the *cps* locus and the rest of the pneumococcal genome that could be inferred from these data was specific to serotype, not serogroup, and no evidence was found that the observed predominance of within-serogroup switches is explained by serogroup-specific adaptations.

### Potential for epistatic interactions

It remains possible that epistasis affects other phenotypes that are not easily assayed in the laboratory; it may be possible to infer these from the genomic data. One factor likely to impact on the range of capsules a strain may successfully express is its complement of carbohydrate transporters [38,39], many of which have been functionally characterised [38] and are only present in a subset of the population (S3 Fig & S3 Table). While capsules in different serogroups usually have distinct chemical compositions, capsules within the same serogroup often consist of the same moieties connected by different bonds. Hence strains may only be adapted to importing and processing certain carbohydrates at high enough rates to sustain capsule production, thereby inhibiting the acquisition of a capsule type with a divergent chemical composition. However, the distribution of transporters across the population does not provide an obvious explanation for the stable association between sequence clusters and serogroups (S3 Fig). SCs 9, 13 and 15 did not appear to have a smaller number of carbohydrate transporters than those in which a change of serogroup had occurred, nor relative to those not having changed serotype. The associated serogroups can be distinguished by the presence of ribitol (serogroup 6), *N*-acetylmannosamine (serogroup 19) and glycerol (serogroup 23). Ribitol is synthesised as part of teichoic acid common to all pneumococci; glycerol is imported by GlpF, encoded by a gene ubiquitous across the sample; and *N*-acetylmannosamine is known to be a substrate for four transporters that are common to all isolates, and one that is absent from SC15. These data do not support the hypothesis that the observed pattern of switching is the result of a constricted ability to acquire the requisite carbohydrate molecules, although epistatic interactions with other aspects of carbohydrate metabolism cannot be excluded.

Yet this analysis only uses experimentally characterised loci, which cannot encompass novel systems that may be rare in the population. It is possible in principle to identify epistatic interactions between any loci from sequence data [40]. In bacteria, interacting accessory genome loci should be present within the same genomes more often than expected by chance, after accounting for linkage and clonal structure. Three sequence clusters, each essentially an independent genotype [41], were universally of serogroup 6 (SC10, SC13 and SC14). A search for clusters of orthologous genes (COGs) that were ubiquitous in these sequence clusters but absent from sequence clusters with no serogroup 6 representatives identified three COGs, all of which corresponded to genes within the serogroup 6 *cps* loci. This failure to identify serogroup 6 associated genes outside the *cps* locus is in keeping with the experimental data that suggests adaptation is specific to a serotype, not a serogroup. Testing for association with serotype requires that the same capsule be acquired multiple times in parallel, as in SC9. Each triallelic polymorphic site in SC9 was tested for association of an allele with one of the three serogroup 23 serotypes. Twelve such sites were identified, all of which were within the *cps* locus. Additionally, no COGs in the accessory genome associated perfectly with any of these individual serotypes. Therefore these simple analyses provided no evidence of sites outside the *cps* locus that might epistatically interact with the expressed capsule type.

A more generic strategy for identifying potential epistatic interactions was not to look for associations of specific sequences with particular *cps* loci, but instead quantify the overall extent of changes accompanying serotype switches. If one or more non-*cps* loci improved the fitness of a novel serotype variant, then it might be expected that serotype switches would be associated with elevated rates of change across the rest of the genome. In order to control for the general heterogeneity in the rates of pneumococcal recombination [36], the total lengths of the recombinations occurring on the same phylogenetic branch as serotype switches (both within- and between-serogroups) were compared with those occurring on branches on which recombinations affected the *cps* locus but did not cause a change in serotype (Fig. 5D). This found that similar levels of contemporaneous (or near-contemporaneous) recombination occurred whether or not there was a change in serotype or serogroup. Additionally, all three types of recombination affecting the *cps* locus were typically associated with sufficient numbers of base substitutions being imported by recombination (S4 Fig) to saturate the mismatch repair system [42]. Hence, there was no significant evidence of non-*cps* loci being exchanged to facilitate the acquisition of a new serotype or serogroup, nor of mismatch repair having an impact on the patterns of serotype switching.

## Discussion

Previous study of this collection of carried pneumococci, based on serotyping and MLST data, identified a borderline-significant tendency for serotype switches to occur within serogroups [21]. The availability of whole genome-based phylogenies increases the number of switches that can be investigated within the same set of isolates through identification of two classes of switch invisible to the MLST-based method: changes in serotype along a lineage in which the multilocus sequence type has also changed (e.g. SC9), and also multiple independent switches within the same sequence type (e.g. in SC13, two switches to 6C within MLST 473). This increases the sample size of identified serotype switching events from nine to twenty. Also, these data provide more reliable phylogenetic information on the ancestral serotype: previously, this was based on the temporal order in which combinations of serotype and genotype were observed, which can be misleading in certain cases, such as SC9 [25].

Future population genomic datasets will determine how general the preponderance of within-serogroup switching is across the pneumococcal species; a recently published dataset of over 3,000 genomes systematically sampled from a Thai refugee camp, in which hosts were unvaccinated, provides an opportunity to test the reproducibility of the pattern [43]. Applying the simple permutation test to the distribution of serotypes between sequence clusters in which switching had occurred, as for the dataset described in this study, revealed the ratio of serogroup concordant to serotype-discordant comparisons to be 0.14 (*p* = 0.0006 from 10,000 permutations). The serotypes in this sample are markedly different from those in the Massachusetts dataset; hence this enrichment of within-serogroup switching provides independent support for the original observation. Additionally, although only one within-serogroup switch was observed in SC15 in this dataset, the acquisition of the 19A capsule in place of the 19F type has occurred at least three further times in related isolates [33].

Multiple hypotheses were proposed as potential explanations for the observed frequency of within-serogroup switches. The first was that the pattern was caused by the constraints of genetic transformation, with short recombination events resulting in partial alteration of the *cps* locus being more frequent. The suggestion that most recombinations are too short to be likely to cause a change in serotype was supported by the large number of ‘silent’ recombinations affecting the *cps* locus, which did not change the antigenic profile of the bacterium. While some cases are likely to reflect exchanges of sequence between distinct isolates of the same serotype, many of these events affect the 5’ or 3’ boundaries of the *cps* locus that are the most strongly conserved across different *cps* loci, and therefore may originate in donors with a very different *cps* locus to that of the recipient. As these exchanges do not result in antigenic changes, it may be that they represent neutral diversification, or serve a role in repairing deleterious mutations. This potential for recombination between serotypes that does not cause switching should be borne in mind when designing sequence-based serotyping methodologies that do not directly target the polymorphisms within *cps* loci that cause differences in capsule type.

Nevertheless, recombinations causing within-serogroup switching sometimes did not span the entire *cps* locus, as all the between-serogroup switches did. In three cases, the recombinations were sufficiently restricted in their extent so as to end within a serogroup-specific part of the *cps* locus; however, these were still substantially larger than the putative minimal genetic changes that could cause the same alteration of serotype. Furthermore, the enrichment for within-serogroup switching remained highly significant even when the events that did not span the entire *cps* locus were excluded; although this does not rule out this mechanism contributing to the observed pattern of switching, this result demonstrates it is not the sole reason underlying it. Finally, the within-serogroup switches did not appear to represent a ‘neutral’ set of typical recombination lengths, as they were still significantly larger than those which caused no alteration of serotype overall, although there may be a limit on the length of such recombinations before they are likely to stop being ‘silent’. One possibility not considered here, subtly different to a dependence on the absolute length of a recombination, is that restriction modification systems could be limiting the transfer of *cps* loci. Although restriction endonucleases typically do not affect transformation, as DNA is imported in a single stranded form, in cases where genomic islands are imported, the synthesis of a complementary strand once the DNA is integrated into the genome can render the acquired locus sensitive to endonucleolysis [44]. Loci imported from a different serogroup would have greater sequence divergence, and therefore a greater set of potential target motifs that could be subject to endonucleolysis. However, this will remain difficult to assess until our knowledge of the diverse set of pneumococcal restriction-modification systems has improved [41].

Conversely, there was no evidence that serotype switching recombinations were selected for the lengths to which they affected the regions flanking the *cps* locus and the *pbp* genes. Co-incidence of serotype switches with changes in β–lactam resistance [32] was rare and there was little evidence of recombinations being constrained in the extent to which they extended into the regions flanking the *cps* locus. However, there are uncertainties associated with ascertaining these recombination breakpoints. One is that the evolutionary time represented by the long branches on which the changes of serogroup occur within SC1 may be sufficient to permit several rounds of transformation. This could result in overlapping recombinations being erroneously inferred to represent a single event, thereby increasing the apparent length of the recombined sequence. However, as both events affected by this bias were between-serogroup switches, this cannot account for within-serogroup switches being longer than the minimum length required for the phenotypic change. Conversely, multiple switches to the same serotype in parallel produce extensive homoplasy. Inaccurate reconstruction of such homoplasies could result in individual actual recombination events being reconstructed as multiple fragmentary events split between different branches of the phylogeny. This risks artificially shortening serotype-switching recombinations. However as this bias only affects sequence clusters in which similar *cps* loci are acquired multiple times, which also happen to be those in which within-serogroup switching is most common (SC9 and SC13), any such errors are conservative with regard to the finding that shorter recombinations alone cannot account for the observed pattern of within-serogroup switching. One potential example is switch 23A→23F**, as the two *cps* loci apparently imported by this recombination are not particularly closely related (S1 Fig); hence this inferred recombination may in fact represent only a partial fragment of two separate serotype switching recombinations. If this were correct, it would increase the frequency of within-serogroup switches further, and suggest that the short recombination currently annotated as the single switch 23A→23F** actually corresponds to two separate, longer events that cannot be resolved with the current collection. This ambiguity highlights how unlikely it is that the numbers of parallel switching events have been overestimated as a consequence of incorrect evolutionary reconstructions. Given the substantial numbers of polymorphisms caused by capsule switching recombinations in the region of the *cps* locus, independent acquisitions in parallel are unlikely to be inferred without strong evidence of distinct ancestry from the rest of the chromosome. Furthermore, the diversity of the *cps* loci themselves, not directly used in the phylogenetic inference displayed in Fig. 3, independently support the same reconstructed pattern of switches occurring in parallel within SC9 and SC13.

As limitations to recombination do not appear to explain the observed pattern of switching, selection for certain combinations of *cps* loci and genetic backgrounds may explain the distribution of capsule across the population. Metabolic or physiological adaptation between the *cps* locus and other aspects of the pneumococcal genotype may result in a limited range of capsule types that can be successfully expressed by a given genomic background. Although serotype itself affects growth rate [45], this study found that the rate of growth *in vitro* depended on both the serotype and backbone genotype in a non-additive way. Isolates grew fastest when expressing their native capsule, although R34-3029 grew similarly fast when expressing either of the tested serogroup 23 capsule loci. However, in most cases there was no difference in the level of growth inhibition when capsules of the same, or a different, serogroup were introduced, particularly when accounting for the growth curves of the donors and recipients involved in each exchange. This is consistent with the existence of epistatic interactions between the *cps* locus and the rest of the genome, but not with the hypothesis that such interactions were serogroup-specific. It could still be that the changes required to adapt to the acquisition of a more similar serotype are relatively small, and more likely to occur through recombination or mutation before the capsule variant is selected out of the population. Yet over the timescales easily testable in the laboratory, no evidence was found for a mechanism that would explain the frequency of within-serogroup switching.

One potential confounding problem with these experiments is the chance that non-*cps* loci could be co-transferred, and therefore influence the fitness of the resulting capsule-switched recombinants. However, previous experiments have suggested that random co-transfer of other sequence should not systematically affect the growth rates of transformants [28]. Furthermore, it might be expected that any co-transfer from the *cps* locus donor might facilitate adaptation to the acquired serotype, if there were epistatic interactions between the *cps* locus and the rest of the genome. However, the genomic data provided no evidence of natural serotype switches being associated with unusually high levels of sequence exchanges around the rest of the genome, suggesting the co-transfer of other loci is not likely to facilitate adaptation to an altered capsule type. Furthermore, there were no signs of non-*cps* loci associated with pneumococci of specific serogroups or serotypes in a manner suggestive of interactions between distinct loci in the same chromosome. Therefore, the genomic and experimental data do not support the model of simple, strong epistatic interactions determining the range of serogroups that an isolate can successfully express.

One longstanding hypothesis for the maintenance of “strain structure” in recombining pathogens is that host immunity produces strains that maintain discordant sets of antigens [34]. Here, individual capsular antigens found across serogroups might be associated with a particular set of subcapsular antigens, creating epistasis in the presence of host immunity that would not be observable *in vitro*. Alternatively, opportunities for recombination may be disproportionately available for strains in the same serogroup as a result of serogroup-wide immunity making hosts either susceptible or resistant to colonization with particular serogroups. Both these mechanisms rely on the assumption that anticapsular antibodies are protective against carriage, and that at least part of this protection is by responses that target epitopes shared across serotypes within a serogroup. Whether this could significantly impact the rate of genetic exchange between serotypes remains an open question. The existence of cross-protection by naturally-acquired anticapsular antibodies within a serogroup is made plausible by the observation of protection against serotype 6A from the 6B component of PCV7, and by the existence of cross-reactive antibodies used in serotyping. However, we are aware of no direct evidence for protection across a serogroup by naturally-acquired anticapsular antibodies. Furthermore, the protective immunity induced by conjugate vaccines is not serogroup-wide, as demonstrated by the post-PCV7 success of some vaccine-related serotypes, such as 19A, 23A, 23B and 6C [16].

Expanded sampling in the future may allow for these and other hypotheses to be tested further, as well as permitting more detailed searches for loci that may interact epistatically with the *cps* locus. Such information regarding which serotypes can be readily ‘interchanged’ may well prove important in understanding the responses to past conjugate vaccine introductions, and predicting the response to future interventions.

## Methods

### Permutation tests

When calculating the significance of the association of serogroups with sequence clusters, the first test resampled each of the serotypes listed in S1 Table without replacement until each sequence cluster had been assigned the same number of serotypes as observed in the actual dataset. This test was therefore independent of the phylogenies and reconstructed patterns of serotype switching. Ten thousand such permutations were carried out, and in each case the calculated test statistic was the ratio of serogroup-concordant to serotype-discordant pairwise comparisons within sequence clusters. Any pairwise comparison between identical serotypes was ignored, as it would be serologically undetectable. While recombinations between isolates of the same serotype at the *cps* locus may be detected in this dataset as a subset of those events that do not alter the expressed capsule type, it is not possible to easily distinguish these from recombinations donated from isolates only of the same serogroup, or of a completely unrelated serotype, which may also not affect the recombinant’s expressed capsule type, depending on how conserved different parts of the *cps* locus are across pneumococci of different serotypes. The *p* value was calculated as the proportion of permutations for which the calculated test statistic was greater than, or equal to, the value observed in the actual population (0.41). When applied to the Maela dataset, the ‘secondary BAPS’ groupings were used as the equivalent of sequence clusters.

The second permutation test used information from the phylogenetic reconstructions of serotype switching. The input dataset was the twenty switches for which the ancestral and derived serotypes could both be defined (Fig. 3). Ten thousand permutations were performed in which the ancestral and derived serotypes were sampled, without replacement, from those in the input dataset. In each case, the test statistic was the proportion of serotype switches that were serogroup concordant, and the *p* value calculated as the proportion of the permutations in which this statistic was equal to, or greater than, the observed value (0.55).

### Reconstruction of serotype changes

The phylogenies used to identify serotype switches have been reported previously [25]. Briefly, a reference genome was assembled *de novo* for each sequence cluster, against which Illumina reads were aligned, and polymorphisms identified [28]. Sequences imported by putative recombination events were identified and removed, and a maximum likelihood phylogeny generated from the remaining clonal frame, as described [29] and validated [46] previously. The annotation of putative mobile genetic elements allowed the number of base substitutions introduced by point mutation, homologous recombination and non-homologous recombination to be estimated. All lengths and positions of recombination events are therefore relative to the reference sequences against which the Illumina reads were originally mapped. The identification of putative homologous recombination events associated with serotype switching is detailed in the supplement S1 Text; this also describes the assessment of within-serogroup sequence diversity, and the reconstruction of changes in serotype, achieved using the maximum likelihood approach implemented in the APE R package [47]. This approach, as well as a maximum parsimony method, was also used to reconstruct the emergence of β–lactam resistance using the trees based on the clonal frame, once each isolate had been classified as either ‘resistant’ (benzylpenicillin minimum inhibitory concentration >0.06 μg mL^-1^) or ‘sensitive’. The details of this analysis are also described in S1 Text and S2 Table.

### Construction of *in vitro* serotype switches

The *cps* locus of isolates was first knocked out using a Janus cassette [48] as described previously [49]. Transformation was conducted using genomic DNA from the serotype donor and the competence stimulating peptide appropriate to the genotype. After 2 h growth, selection was performed using plates containing 500 μg ml^-1^ streptomycin. Colonies were replica plated to ensure loss of kanamycin resistance and expression of the altered serotype confirmed through latex agglutination (Statens Serum Institut, Copenhagen).

### Comparisons of growth

Isolates were grown to mid-log phase in THY (Todd Hewitt broth containing 0.5% yeast extract), titered and frozen at -70°C in 10% glycerol. Growth was compared in parallel by inoculating 6 mL of THY with a starting concentration of 10^6^ mL^-1^ bacteria from freshly thawed frozen stock. Cultures were incubated at 37°C with 5% CO_2_. At successive time points 200 μL was removed to a 96 well plate. Optical density at a wavelength of 610 nm was measured and dilutions were plated on blood agar plates to calculate live cell densities.

## Supporting Information

S1 Fig Diversity of (A) serogroup 23 *cps* loci within SC9, as compared to all serogroup 23 *cps* loci in the collection, and (B) serogroup 6 *cps* loci within SC13, as compared to all serogroup 6 *cps* loci in the collection. The *cps* loci derived from individual serotype switching events are annotated as in Fig. 3. All switches occurring in parallel imported distinct loci, indicating they are genuinely separate events. All *cps* loci derived from individual switches were monophyletic, suggesting the inferred reconstruction was correct, with the exception of switch 23A->23F**. In this case, the two *cps* loci were polyphyletic, indicating this was a single capsule switch that was followed by subsequent diversification through recombinations at the *cps* locus that did not alter serotype (as indicated by Fig. 3), or two capsule switches to 23F occurring in parallel in closely-related isolates. This latter conclusion would further strengthen the observed enrichment for within-serogroup switching.
(PDF)

S2 Fig Growth curves of isolates used as recipients and donors of *cps* loci in the capsule switching experiments. All plots show the number of viable cells at different sampling times as colony forming units per millilitre. Solid lines indicate isolates expressing the dominant serogroup in a sequence cluster; point styles match those in Fig. 6. (A) Growth curves of recipient isolates from SC9 and the donors of the serotype 6B and 18C *cps* loci. Three replicates are shown in plots (i)-(iii). (B) Growth curves of recipient isolates from SC13 and the donor of the 15F capsule type. Three replicates are again shown in plots (i)-(iii).
(PDF)

S3 Fig Distribution of carbohydrate transporters across the population. (A) Maximum likelihood phylogeny, as displayed in Fig. 1. (B) Characterised carbohydrate transporters labelled with the gene names or the corresponding locus tag codes in *S*. *pneumoniae* TIGR4 [EMBL accession code: AE005672] or ATCC 700669 [EMBL accession code: FM211187]. Alternating orange and brown boxes indicate the individual COG sequences that comprise each transport system. Solid vertical black lines divide the sequences associated with different transport systems. Dashed vertical black lines divide the sequences associated with alternative alleles of the same locus. The likely substrates of each transporter are listed in S3 Table. (C) Red cells indicate the presence of the COG at the top of the column in the isolate specified by the phylogeny; blue cells indicate the COG is absent from the isolate.
(PDF)

S4 Fig Number of base substitutions imported by recombinations outside of the *cps* locus. In each boxplot, the recombinations affecting the *cps* locus are grouped according to their inferred impact on serotype. (A) The number of base substitutions outside the *cps* locus introduced by recombinations overlapping with the *cps* locus. Values of zero indicate the recombination entirely lay within the *cps* locus; values greater than zero reflect the extent to which the recombination extended into the regions flanking the *cps* locus. (B) The number of base substitutions outside the *cps* locus introduced by recombinations occurring on the same branch of the phylogeny as a recombination overlapping with the *cps* locus. This shows the genetic diversity imported by recombinations that are likely to be contemporaneous, or near-contemporaneous, with a recombination affecting the *cps* locus.
(PDF)

S1 Table List of serotype diversity within sequence clusters used in the permutation tests. (DOCX)

S2 Table Linkage of serotype switches and changes in β–lactam resistance. Recombinations affecting the *cps* locus are listed in the same order as in Fig. 4, with the exception that those occurring on the same branch of the phylogeny were merged into a single row in this table. The changes in phenotype along the corresponding branch, in terms of capsule and β–lactam resistance profile (as estimated by both maximum likelihood and maximum parsimony approaches), are detailed.
(DOCX)

S3 Table Functional characteristics of carbohydrate transporters. Each transport system is labelled with the relevant gene name, locus tag from *S*. *pneumoniae* TIGR4, or locus tag from *S*. *pneumoniae* ATCC 700669. The putative substrates are summarised from Bidossi *et al*.
(DOCX)

S1 Text Reconstruction of serotype switches. (DOCX)

S2 Text Detail of individual changes in β–lactam resistance. (DOCX)
