# Supplementary figures and images for: Selective and Genetic Constraints on Pneumococcal Serotype Switching
Source: PLoS Genet. 2015 Mar 31;11(3):e1005095. doi: 10.1371/journal.pgen.1005095 (PMC4380333; doi:10.1371/journal.pgen.1005095)

(A)

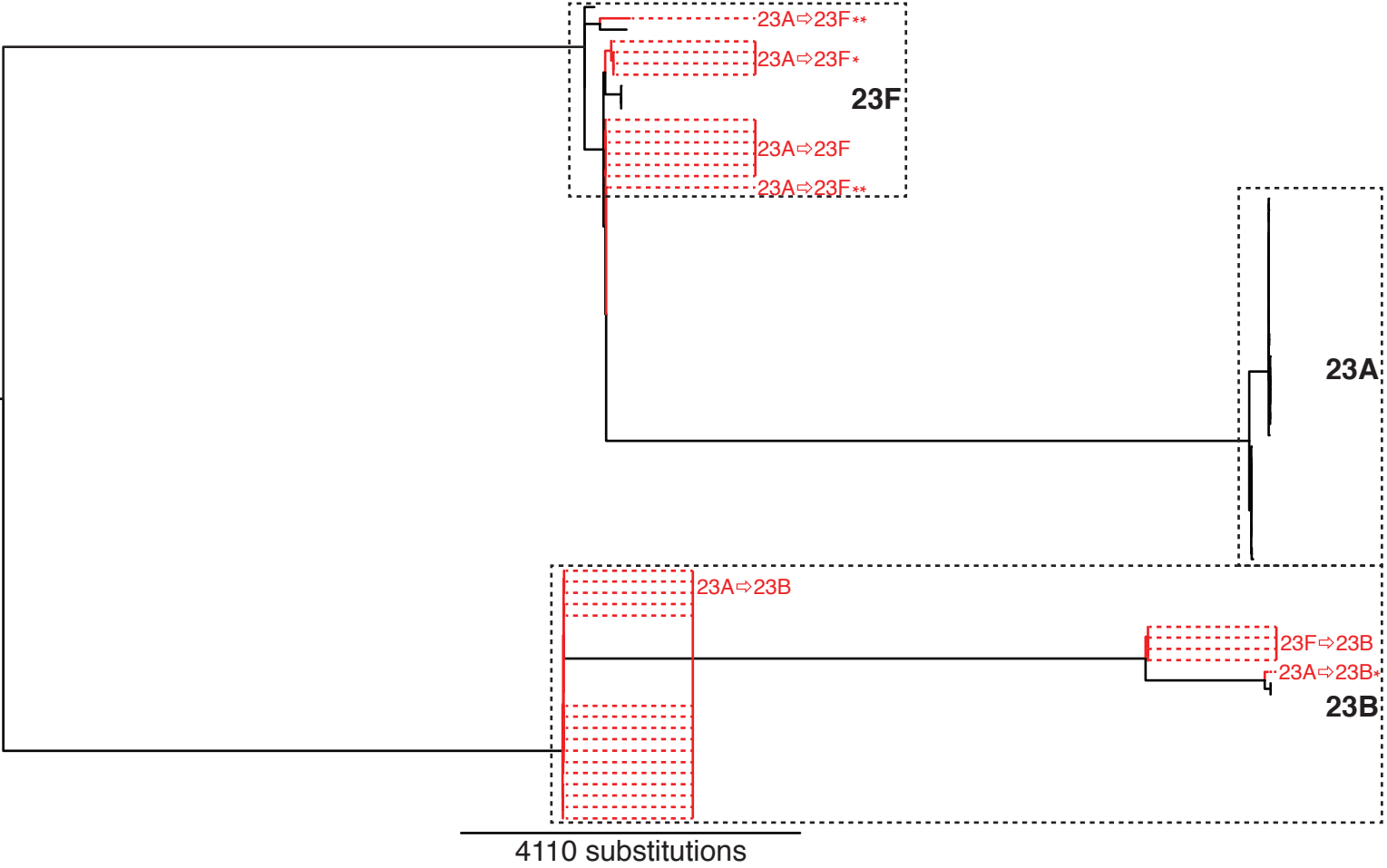

(B)

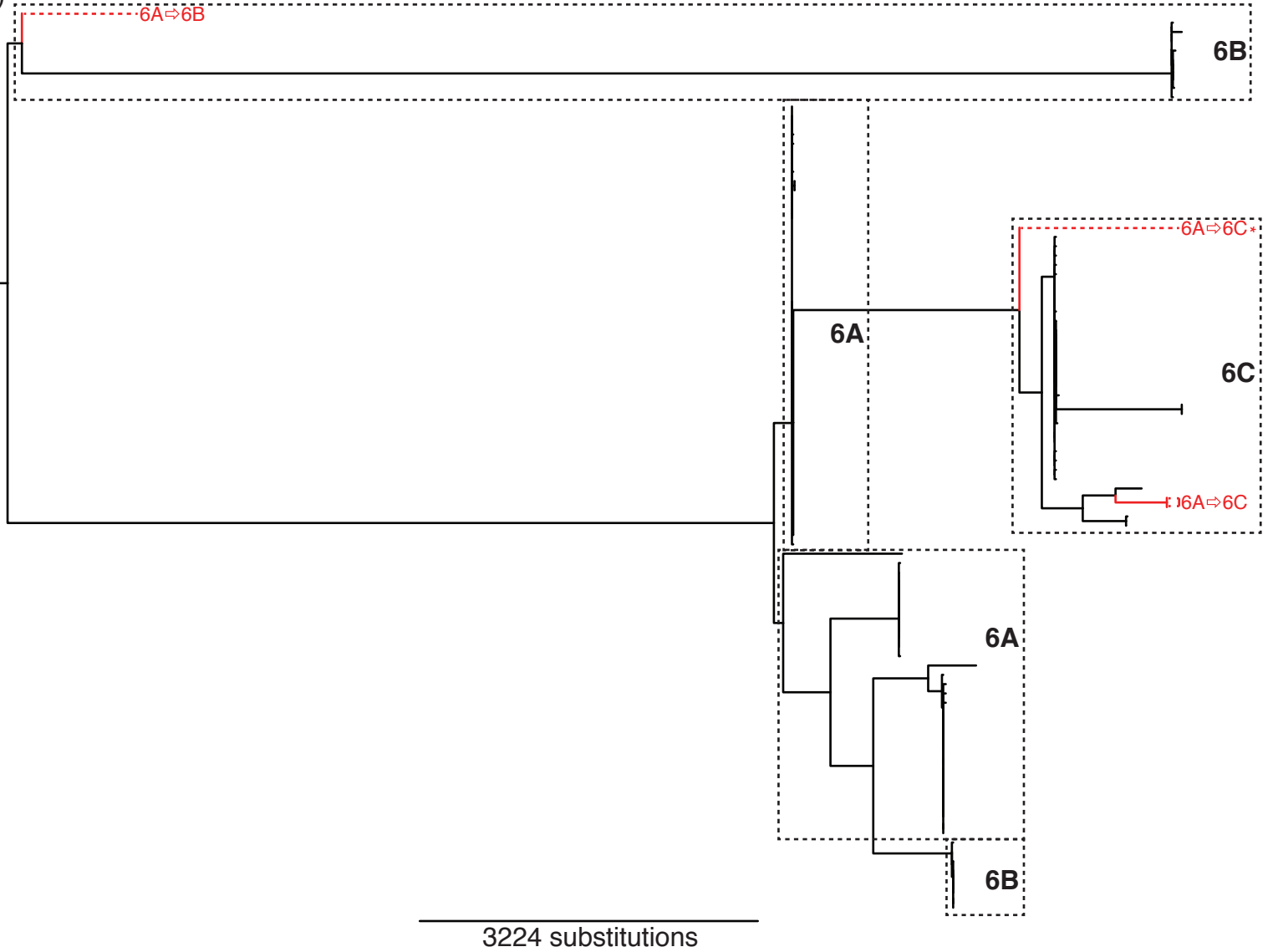

Supplement: S1 Fig — The cps loci derived from individual serotype switching events are annotated as in Fig. 3. All switches occurring in parallel imported distinct loci, indicating they are genuinely separate events. All cps loci derived from individual switches were monophyletic, suggesting the inferred reconstruction was correct, with the exception of switch 23A->23F**. In this case, the two cps loci were polyphyletic, indicating this was a single capsule switch that was followed by subsequent diversification through recombinations at the cps locus that did not alter serotype (as indicated by Fig. 3), or two capsule switches to 23F occurring in parallel in closely-related isolates. This latter conclusion would further strengthen the observed enrichment for within-serogroup switching. (PDF) [file pgen.1005095.s001.pdf]

(A) (i)

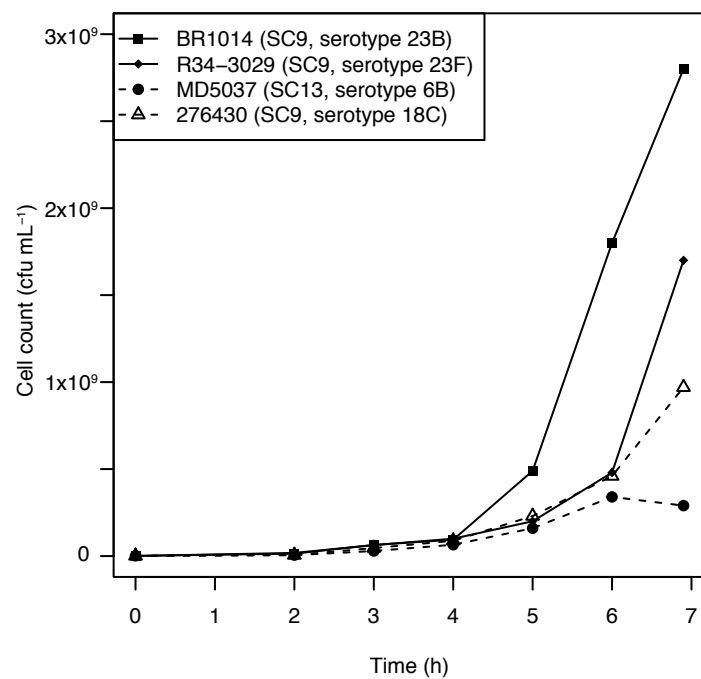

(ii)

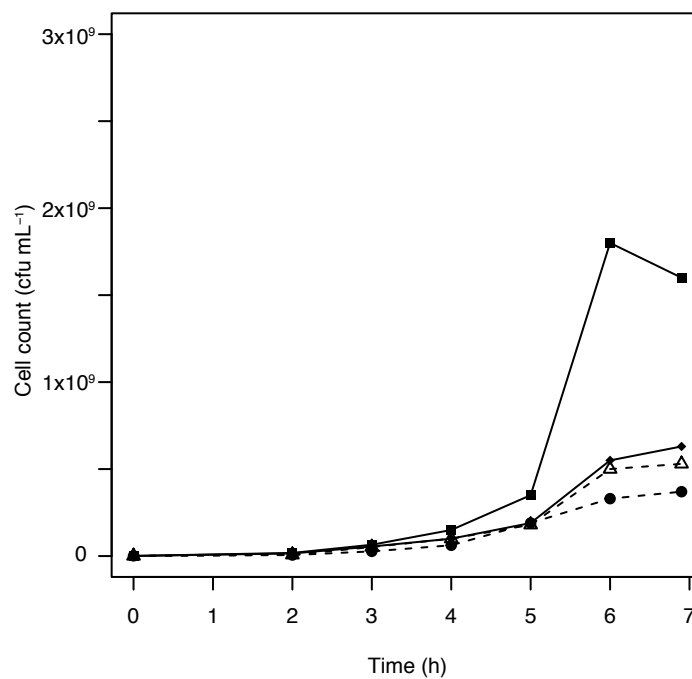

(iii)

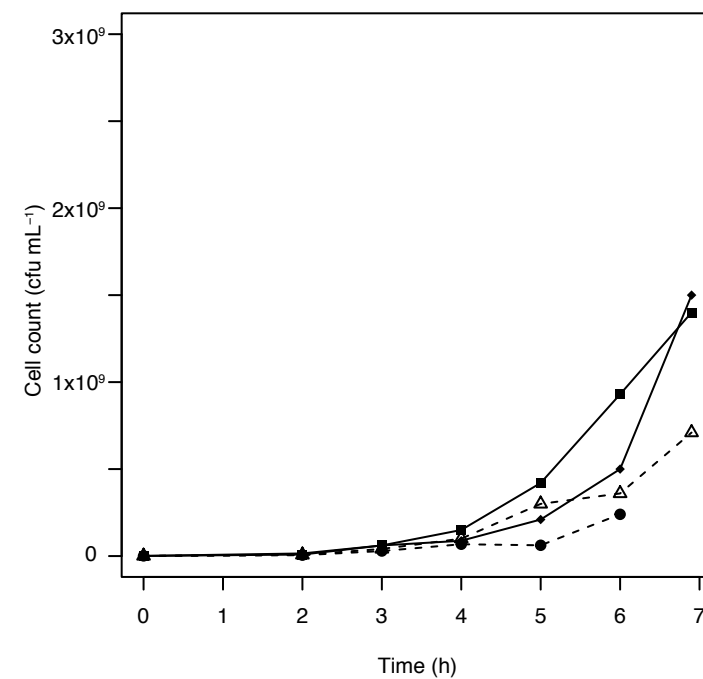

(B) (i)

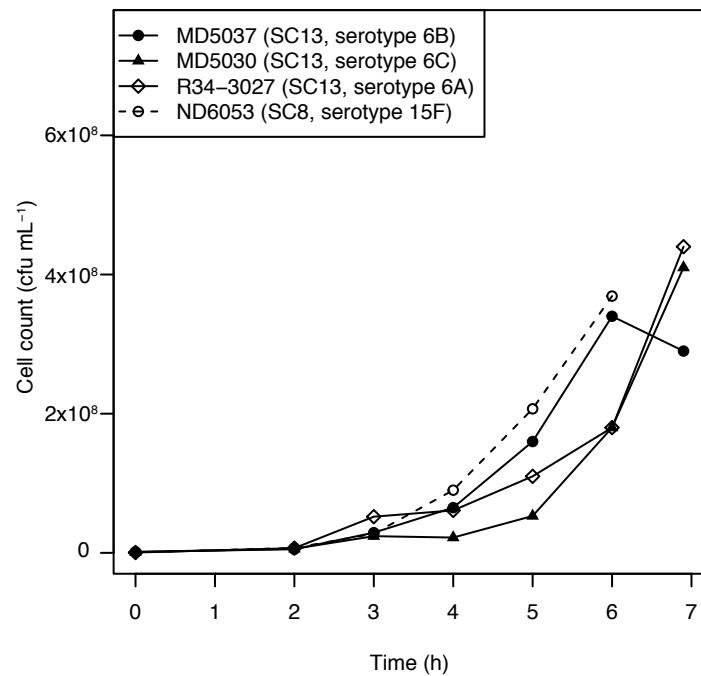

(ii)

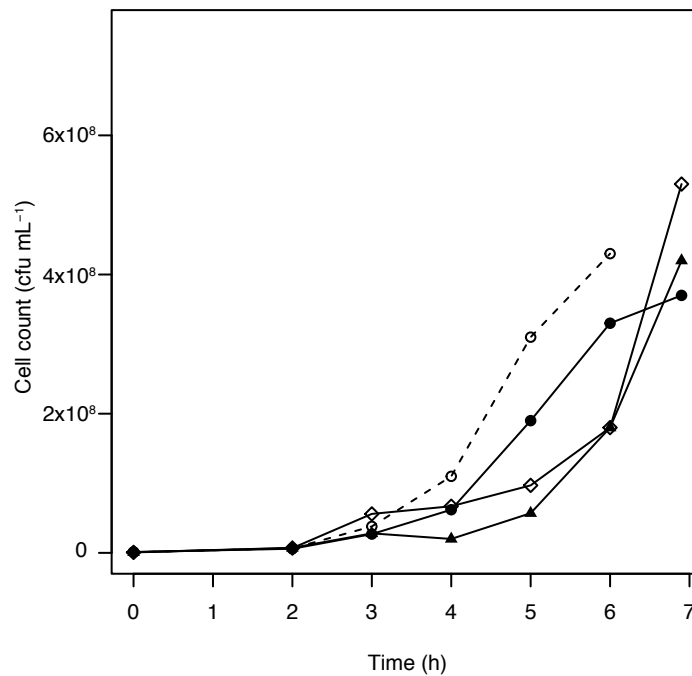

(iii)

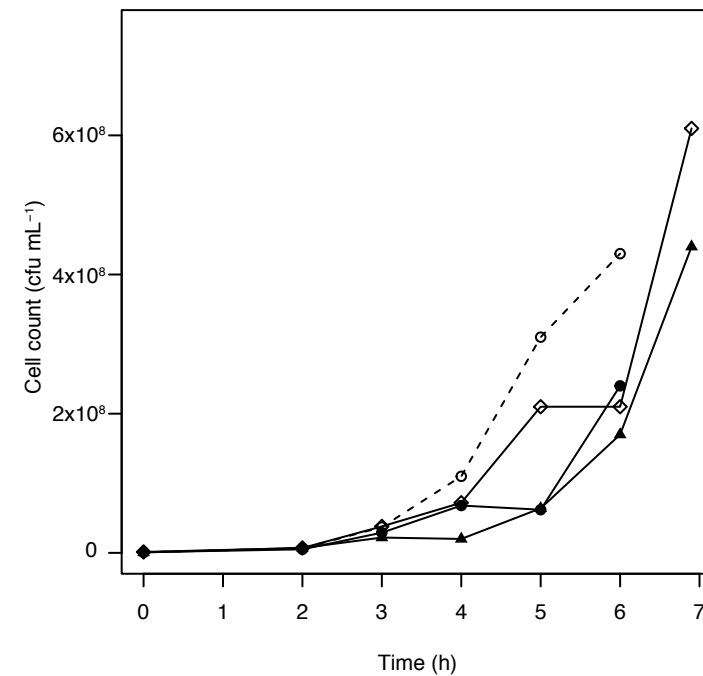

Supplement: S2 Fig — All plots show the number of viable cells at different sampling times as colony forming units per millilitre. Solid lines indicate isolates expressing the dominant serogroup in a sequence cluster; point styles match those in Fig. 6. (A) Growth curves of recipient isolates from SC9 and the donors of the serotype 6B and 18C cps loci. Three replicates are shown in plots (i)-(iii). (B) Growth curves of recipient isolates from SC13 and the donor of the 15F capsule type. Three replicates are again shown in plots (i)-(iii). (PDF) [file pgen.1005095.s002.pdf]

S3 Figure

(A)

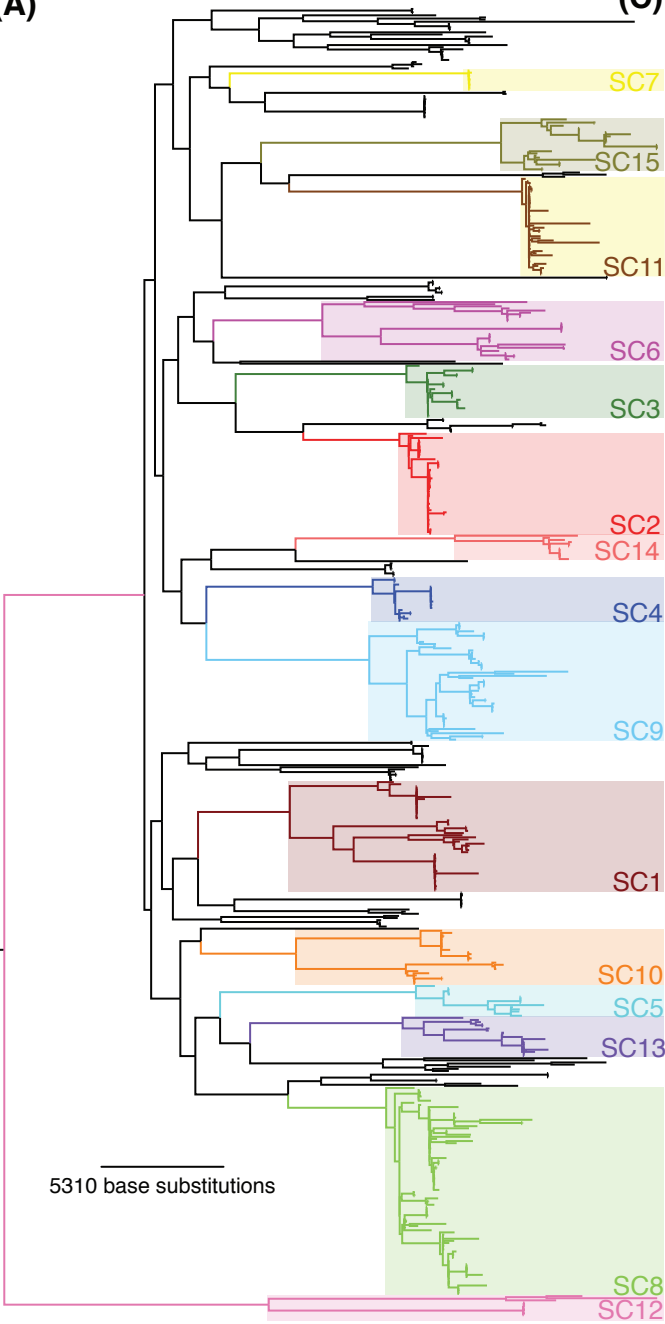

Key

- Present in assembly
- Absent from assembly

(B)

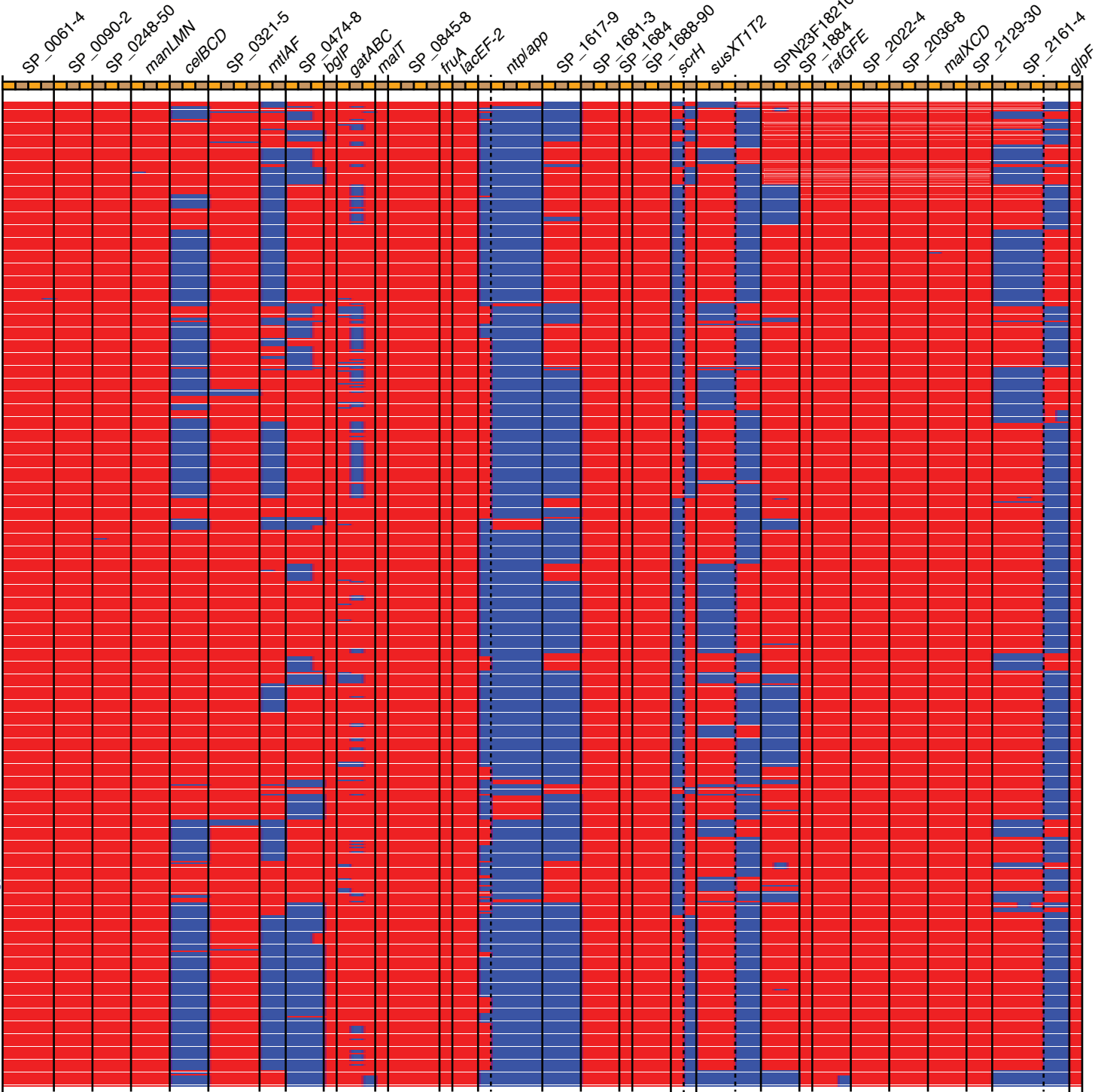

(C)

Supplement: S3 Fig — (A) Maximum likelihood phylogeny, as displayed in Fig. 1. (B) Characterised carbohydrate transporters labelled with the gene names or the corresponding locus tag codes in S. pneumoniae TIGR4 [EMBL accession code: AE005672] or ATCC 700669 [EMBL accession code: FM211187]. Alternating orange and brown boxes indicate the individual COG sequences that comprise each transport system. Solid vertical black lines divide the sequences associated with different transport systems. Dashed vertical black lines divide the sequences associated with alternative alleles of the same locus. The likely substrates of each transporter are listed in S3 Table. (C) Red cells indicate the presence of the COG at the top of the column in the isolate specified by the phylogeny; blue cells indicate the COG is absent from the isolate. (PDF) [file pgen.1005095.s003.pdf]

S4 Figure

(A)

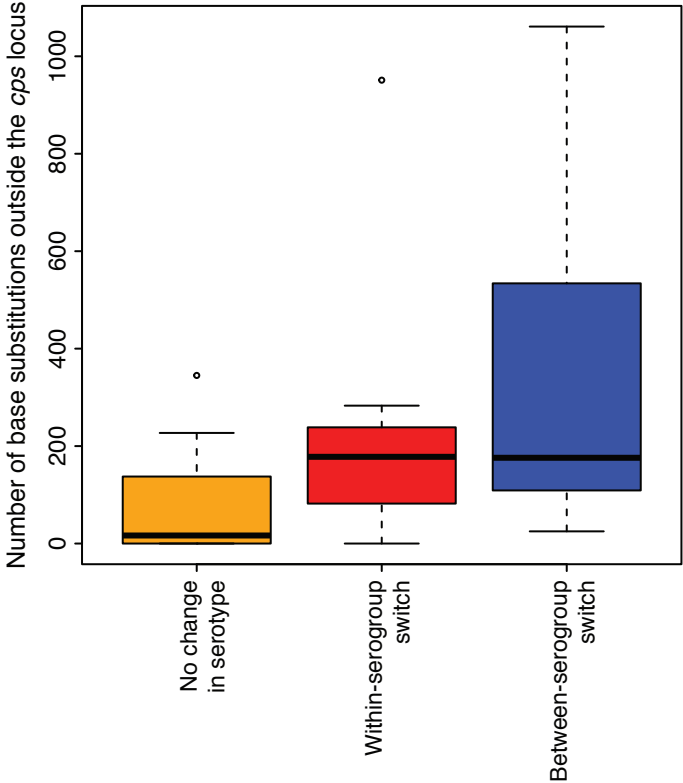

(B)

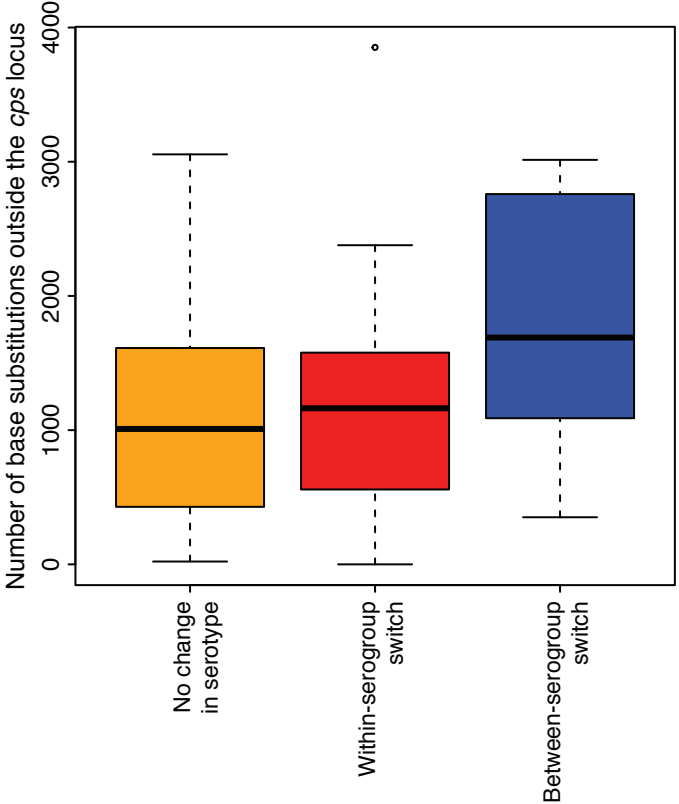

Supplement: S4 Fig — In each boxplot, the recombinations affecting the cps locus are grouped according to their inferred impact on serotype. (A) The number of base substitutions outside the cps locus introduced by recombinations overlapping with the cps locus. Values of zero indicate the recombination entirely lay within the cps locus; values greater than zero reflect the extent to which the recombination extended into the regions flanking the cps locus. (B) The number of base substitutions outside the cps locus introduced by recombinations occurring on the same branch of the phylogeny as a recombination overlapping with the cps locus. This shows the genetic diversity imported by recombinations that are likely to be contemporaneous, or near-contemporaneous, with a recombination affecting the cps locus. (PDF) [file pgen.1005095.s004.pdf]
