# Supplementary material for: Selective and Genetic Constraints on Pneumococcal Serotype Switching
Source: PLoS Genet. 2015 Mar 31;11(3):e1005095. doi: 10.1371/journal.pgen.1005095 (PMC4380333; doi:10.1371/journal.pgen.1005095)
Supplement: S1 Table — (DOCX) [file pgen.1005095.s005.docx]

**S1 Table** List of serotype diversity within sequence clusters used in permutation tests.

| **Sequence Cluster** | **Serotypes** |
| --- | --- |
| 1 | 6A, 10A, 35F |
| 3 | 15A, 19A |
| 5 | 9V, 11A, 15B/C, 19A |
| 6 | 6B, 6C, 15B/C, 23A, 23B, 23F |
| 8 | 7C, 15B/C, 19A |
| 9 | 18C, 23A, 23B, 23F |
| 13 | 6A, 6B, 6C |
| 15 | 19A, 19F |
