# Supplementary material for: Selective and Genetic Constraints on Pneumococcal Serotype Switching
Source: PLoS Genet. 2015 Mar 31;11(3):e1005095. doi: 10.1371/journal.pgen.1005095 (PMC4380333; doi:10.1371/journal.pgen.1005095)
Supplement: S2 Table — Recombinations affecting the cps locus are listed in the same order as in Fig. 4, with the exception that those occurring on the same branch of the phylogeny were merged into a single row in this table. The changes in phenotype along the corresponding branch, in terms of capsule and β–lactam resistance profile (as estimated by both maximum likelihood and maximum parsimony approaches), are detailed. (DOCX) [file pgen.1005095.s006.docx]

**S2 Table** Linkage of serotype switches and changes in β–lactam resistance. Recombinations affecting the *cps* locus are listed in the same order as in Fig 4, with the exception that those occurring on the same branch of the phylogeny were merged into a single row in this table. The changes in phenotype along the branch, in terms of capsule and β–lactam resistance profile (as estimated by both maximum likelihood and maximum parsimony approaches), are detailed.

| **SC** | **Affect on Serotype** | **Affects *pbp2x*?** | **Affects *pbp1a*?** | **Maximum likelihood β–lactam susceptibility phenotype reconstruction** | | **Maximum parsimony β–lactam susceptibility phenotype reconstruction** | |
| --- | --- | --- | --- | --- | --- | --- | --- |
|  |  |  |  | **Ancestral** | **Derived** | **Ancestral** | **Derived** |
| 1 | 10A->10A | YES | NO | Sensitive | Sensitive | Sensitive | Sensitive |
| 1 | 10A->10A | YES | NO | Sensitive | Sensitive | Sensitive | Sensitive |
| 1 | 10A->10A | YES | NO | Sensitive | Sensitive | Sensitive | Sensitive |
| 1 | 10A->10A | NO | NO | Sensitive | Sensitive | Sensitive | Sensitive |
| 1 | 10A->35F | NO | NO | Sensitive | Sensitive | Sensitive | Sensitive |
| 1 | 10A->6A | NO | NO | Sensitive | Sensitive | Sensitive | Sensitive |
| 3 | 15A->19A | YES | YES | Resistant | Resistant | Resistant | Resistant |
| 5 | 9V->11A | YES | YES | Resistant | Resistant | Resistant | Resistant |
| 5 | 9V->9V | YES | NO | Resistant | Resistant | Resistant | Resistant |
| 5 | 9V->19A | YES | YES | Resistant | Resistant | Resistant | Resistant |
| 5 | 9V->9V | NO | NO | Sensitive | Sensitive | Sensitive | Sensitive |
| 5 | 9V->15B/C | NO | NO | Sensitive | Sensitive | Sensitive | Sensitive |
| 6 | 6B->6C | YES | NO | Sensitive | Sensitive | Sensitive | Sensitive |
| 8 | 15B/C->15B/C | YES | NO | Sensitive | Sensitive | Sensitive | Sensitive |
| 8 | 15B/C->7C | YES | YES | Sensitive | Sensitive | Sensitive | Sensitive |
| 8 | 15B/C->19A | YES | YES | Sensitive | Sensitive | Sensitive | Sensitive |
| 8 | 15B/C->15B/C | NO | NO | Sensitive | Sensitive | Sensitive | Sensitive |
| 8 | 15B/C->15B/C | NO | NO | Sensitive | Sensitive | Sensitive | Sensitive |
| 8 | 15B/C->15B/C | NO | NO | Sensitive | Resistant | Sensitive | Sensitive |
| 8 | 15B/C->15B/C | NO | NO | Resistant | Resistant | Resistant | Resistant |
| 8 | 15B/C->15B/C | NO | YES | Sensitive | Sensitive | Sensitive | Sensitive |
| 8 | 15B/C->15B/C | NO | YES | Sensitive | Sensitive | Sensitive | Sensitive |
| 9 | 23A->23F | YES | NO | Sensitive | Sensitive | Sensitive | Sensitive |
| 9 | 23A->23B | YES | YES | Sensitive | Sensitive | Sensitive | Sensitive |
| 9 | 23A->23A | YES | NO | Sensitive | Sensitive | Sensitive | Sensitive |
| 9 | 23A->23F | YES | YES | Sensitive | Sensitive | Sensitive | Sensitive |
| 9 | 23A->23B | YES | NO | Sensitive | Sensitive | Sensitive | Sensitive |
| 9 | 23B->23B | NO | NO | Sensitive | Sensitive | Sensitive | Sensitive |
| 9 | 23A->23A | NO | NO | Sensitive | Sensitive | Sensitive | Sensitive |
| 9 | 23A->18C | NO | NO | Sensitive | Sensitive | Sensitive | Sensitive |
| 9 | 23A->23A | NO | NO | Sensitive | Sensitive | Sensitive | Sensitive |
| 9 | 23F->23B | NO | YES | Sensitive | Sensitive | Sensitive | Sensitive |
| 9 | 23F->23F | NO | NO | Sensitive | Resistant | Sensitive | Sensitive |
| 9 | 23A->23F | NO | NO | Sensitive | Sensitive | Sensitive | Sensitive |
| 9 | 23F->23F | NO | NO | Resistant | Resistant | Sensitive | Resistant |
| 9 | 23F->23F | NO | NO | Resistant | Sensitive | Sensitive | Sensitive |
| 9 | 23A->23A | NO | NO | Sensitive | Sensitive | Sensitive | Sensitive |
| 9 | 23A->23A | NO | NO | Sensitive | Sensitive | Sensitive | Sensitive |
| 9 | 23F->23F | NO | YES | Sensitive | Resistant | Ambiguous | Resistant |
| 13 | 6A->6C | YES | NO | Sensitive | Sensitive | Ambiguous | Sensitive |
| 13 | 6A->6C | YES | NO | Resistant | Sensitive | Ambiguous | Sensitive |
| 13 | 6A->6B | NO | NO | Sensitive | Resistant | Resistant | Resistant |
| 15 | 19F->19A | YES | NO | Resistant | Resistant | Resistant | Resistant |
| 15 | 19F->19F | NO | NO | Resistant | Resistant | Resistant | Resistant |
