# Supplementary material for: Selective and Genetic Constraints on Pneumococcal Serotype Switching
Source: PLoS Genet. 2015 Mar 31;11(3):e1005095. doi: 10.1371/journal.pgen.1005095 (PMC4380333; doi:10.1371/journal.pgen.1005095)
Supplement: S3 Table — Each transport system is labelled with the relevant gene name, locus tag from S. pneumoniae TIGR4, or locus tag from S. pneumoniae ATCC 700669. The putative substrates are summarised from Bidossi et al. (DOCX) [file pgen.1005095.s007.docx]

**S3 Table** Functional characteristics of carbohydrate transporters. Each transport system is labelled with the relevant gene name, locus tag from *S. pneumoniae* TIGR4, or locus tag from *S. pneumoniae* ATCC 700669. The putative substrates are summarised from Bidossi *et al*.

| **Name** | **Alleles** | **COG** | **Likely substrate(s)** |
| --- | --- | --- | --- |
| SP_0061-4 | 1 | CLS00140, CLS00139, CLS00141, CLS00142 | galactose |
| SP_0090-2 | 1 | CLS00160, CLS00159, CLS00161 | galactose, mannose, N-acetylmannosamine |
| SP_0248-50 | 1 | CLS00289, CLS00287, CLS00288 | - |
| *manLMN* | 1 | CLS00319, CLS00320, CLS00318 | glucose, mannose, galactose, fructose, N-acetylglucosamine, glucosamine |
| *celBCD* | 1 | CLS02393, CLS02395, CLS02391 | cellobiose, gentiobiose, arbutin, beta-glucosides, amygdalin, aesculin |
| SP_0321-5 | 1 | CLS00345, CLS00344, CLS00342, CLS00346 | hyaluronic acid, sulphated glycosaminoglycans |
| *mtlAF* | 1 | CLS02016, CLS02014 | mannitol |
| SP_0474-8 | 1 | CLS01953, CLS01951, CLS01949 | mannose |
| *bglP* | 1 | CLS00539 | 1-O-methyl-beta-glucose, beta-glucosides |
| *gatABC* | 1 | CLS00593, CLS00594, CLS00595 | galactose, lactose, galactitol |
| *malT* | 1 | CLS00691 | maltose, maltotriose, maltodextrin, glycogen |
| SP_0845-8 | 1 | CLS00766, CLS00767, CLS00768, CLS00769 | ribonucleosides |
| *fruA* | 1 | CLS00796 | fructose |
| *lacEF-2* | 1 | CLS01042, CLS01043 | lactulose, lactose, tagatose |
| *ntp*/*app* | 2 | CLS02840, CLS02841, CLS02838, CLS02839, CLS01162 | sialic acid, N-acetylmannosamine, aminosugars |
| SP_1617-9 | 1 | CLS02437, CLS02436, CLS02435 | pentoses |
| SP_1681-3 | 1 | CLS01442, CLS01443, CLS01441 | sialic acid, N-acetylmannosamine |
| SP_1684 | 1 | CLS01444 | glucosamine |
| SP_1688-90 | 1 | CLS01447, CLS01446, CLS01448 | sialic acid, N-acetylmannosamine |
| *scrH* | 2 | CLS01478, CLS02553 | sucrose |
| *susXT1T2* | 2 | CLS01546, CLS01547, CLS02661, CLS02660, CLS01548 | sucrose |
| SPN23F18210-30 | 1 | CLS01558, CLS01559, CLS01560 | sulphated glycans |
| SP_1884 | 1 | CLS01632 | trehalose |
| *rafGFE* | 1 | CLS01646, CLS01644, CLS01645 | stachyose, raffinose, melibiose, alpha-galactosides |
| SP_2022-4 | 1 | CLS01749, CLS01748, CLS01747 | amygdalin, beta-glucosides |
| SP_2036-8 | 1 | CLS01761, CLS01762, CLS01760 | ascorbate |
| *malXCD* | 1 | CLS01826, CLS01828, CLS01827 | maltotriose, maltodextrin, glycogen, oligosaccharides |
| SP_2129-30 | 1 | CLS01846, CLS01847 | pentoses |
| SP_2161-4 | 2 | CLS01874, CLS01872, CLS01875, CLS02656, CLS02658, CLS01873 | fucose, L-arabinose |
| *glpF* | 1 | CLS00013 | glycerol |
