# Supplementary material for: Selective and Genetic Constraints on Pneumococcal Serotype Switching
Source: PLoS Genet. 2015 Mar 31;11(3):e1005095. doi: 10.1371/journal.pgen.1005095 (PMC4380333; doi:10.1371/journal.pgen.1005095)
Supplement: S1 Text — (DOCX) [file pgen.1005095.s008.docx]

**S1 Text: Reconstruction of serotype switches**

Overall, 59 putative homologous recombination events overlapped with the *cps* loci annotated in the reference sequences of the fifteen monophyletic sequence clusters [[1](#_ENREF_1)]. However, the eight recombinations associated with SC6 clearly reflected the difficulty of reconstructing the history of a diverse locus across a deep-branching clade, and therefore these were excluded from the dataset and subsequently replaced with a single recombination reflecting the only serotype switch that could be robustly inferred, that in which a 6B *cps* locus was replaced by a 6C *cps* locus (see below). One recombination within the SC9 dataset was entirely encompassed within a longer recombination that occurred on the same branch of the phylogeny; in this case, the shorter recombination was discarded, and only the longer event used in the described analyses. In the case of SC5, the serotype 11A isolate was used as the reference against which all the other sequences were mapped. This resulted in a ‘reference bias’ in the sequence reconstruction that split the recombination that imported the 11A *cps* locus into SC5 into two separate recombinations that flanked, but did not span, the *cps* locus. Therefore these events were merged to give a more accurate reconstruction of this sequence cluster’s evolution. This produced a final dataset of 50 recombinations affecting the *cps* locus, as displayed in Fig 4, spread across 44 different phylogenetic branches.

The branch lengths of the phylogenies were altered using Bioperl [[2](#_ENREF_2)] to either one, if a recombination affected the *cps* locus on the branch, or zero. The serotypes were reconstructed as discrete traits on these modified phylogenies using the discrete states “Equal Rates” model implemented in the R package APE [[3](#_ENREF_3)]. However, this initial reconstruction did not take into account the boundaries of the recombinations affecting the *cps* locus in each case. Therefore the reconstruction was combined with the known genetic differences between serotypes in order to manually edit the reconstruction to be biologically plausible. In SC1, this suggested that the ancestral serotype was 10A, as only the changes basal to the clades of serotype 6A and 35F isolates were long enough to cause a change in serotype. For analogous reasons, in SC5 the ancestral serotype was reconstructed as 9V.

In SC9, there was ambiguity in the case of a clade composed of two sister serotype 23F isolates and a single serotype 18C isolate. The reconstruction of discrete states suggested that the 18C capsule type was acquired first, then replaced in the pair of 23F isolates by a sequential change in serotype. However, the recombination ancestral to both the 18C isolate and clade of 23F isolates, which would cause a between-serogroup switch from the ancestral 23A serotype to 18C in this scenario, did not extend as far as *wzx* at its 3’ boundary. As the 3’ region of 18C *cps* locus is highly distinct from that of the serogroup 23 *cps* loci [[4](#_ENREF_4)], this reconstruction appears incorrect. Instead, it seems likely that this recombination resulted in the alteration to serotype 23F directly from the 23A serotype, as the corresponding *cps* loci are highly similar in their 3’ region [[4](#_ENREF_4)]. Furthermore, the recombination affecting the *cps* locus that is private to the 18C isolate, which spans all of the genes involved in capsule biosynthesis (Fig 4), seemed more likely to cause the between-serogroup switch. Hence the reconstruction was altered from a case in which there were two between-serogroup switches (23A->18C, followed by 18C->23F) to one in which there was one between-serogroup, and one within-serogroup, switch (23A->23F, followed by 23F->18C).

In the case of SC6, the discrete state reconstruction was only able to fully define a single serotype switch, that of 6B to 6C. However, no biologically plausible recombination could be assigned to this alteration. It seemed likely that the reason that the boundaries of the serotype switching recombination could not be accurately determined was that the reference sequence against which reads were mapped was not of serogroup 6. To facilitate more extensive mapping across the serogroup 6 *cps* locus, a new whole genome alignment was generated using a serotype 6B isolate as a reference sequence. The reconstruction produced through the analysis of this new alignment generated a very similar overall phylogeny and pattern of predicted recombinations to the original analysis, displayed in Fig 3; however, it allowed the boundaries of the serotype switching recombination that imported the 6C capsule type to be defined.

These analyses therefore allowed all twenty of the robustly-inferred serotype switches to be attributed to a putative homologous recombination that spanned the genetic locus known to be responsible for differentiating the ancestral and derived serotypes in each switch.

To independently test whether the apparent acquisition of the same capsule types in parallel within SC9 and SC13 were likely to genuinely represent separate events rather than artefacts of the method used for phylogenetic reconstruction, the diversity of serogroups 23 and 6 were assessed independently of the whole genome alignments. The *cps* loci from all serogroup 23 and 6 isolates across the full collection of isolates were extracted from the previously described *de novo* assemblies [[1](#_ENREF_1)] and aligned using progressiveMauve [[5](#_ENREF_5)]. The polymorphic sites were extracted in order to construct a maximum likelihood phylogeny with RAxML [[6](#_ENREF_6)] using a GTR+gamma substitution model with four rate categories. The *cps* loci resulting from the different serotype switching events were then annotated on this phylogeny (S1 Fig), and found to provide evidence for parallel acquisition of non-identical *cps* loci (that nevertheless encoded the genes for the synthesis of the same capsule) that was congruent with the reconstructions displayed in Fig 3.

**Supplementary References**

1. Croucher NJ, Finkelstein JA, Pelton SI, Mitchell PK, Lee GM, et al. (2013) Population genomics of post-vaccine changes in pneumococcal epidemiology. Nature genetics 45: 656-663.

2. Stajich JE, Block D, Boulez K, Brenner SE, Chervitz SA, et al. (2002) The Bioperl toolkit: Perl modules for the life sciences. Genome Res 12: 1611-1618.

3. Paradis E, Claude J, Strimmer K (2004) APE: analyses of phylogenetics and evolution in R language. Bioinformatics 20: 289-290.

4. Bentley SD, Aanensen DM, Mavroidi A, Saunders D, Rabbinowitsch E, et al. (2006) Genetic analysis of the capsular biosynthetic locus from all 90 pneumococcal serotypes. PLoS Genet 2: e31.

5. Darling AE, Mau B, Perna NT (2010) progressiveMauve: multiple genome alignment with gene gain, loss and rearrangement. PLoS One 5: e11147.

6. Stamatakis A, Ludwig T, Meier H (2005) RAxML-III: a fast program for maximum likelihood-based inference of large phylogenetic trees. Bioinformatics 21: 456-463.
