# Supplementary material for: Selective and Genetic Constraints on Pneumococcal Serotype Switching
Source: PLoS Genet. 2015 Mar 31;11(3):e1005095. doi: 10.1371/journal.pgen.1005095 (PMC4380333; doi:10.1371/journal.pgen.1005095)
Supplement: S2 Text — (DOCX) [file pgen.1005095.s009.docx]

**S2 Text – Detail of individual changes in β–lactam resistance**

The seven recombinations that affected both *pbp1a* and *pbp2x* all caused a change of serotype, but did not substantially alter isolates’ β–lactam resistance (S2 Table). For instance, the import of the 19A capsule into both SC3 and SC5 spanned both *pbp1a* and *pbp2x*, but in both cases the strains remained β–lactam resistant. By contrast, when both genes were apparently imported along with the acquisition of the 19A capsule by SC8, this ancestrally β–lactam sensitive genotype did not appear to develop detectably elevated resistance contemporaneously.

Nineteen of the recombinations spanned at least part of *pbp2x*, of which six had no effect on serotype. Only one serotype switching recombination that affected *pbp2x*, an acquisition of the 6C capsule, was potentially associated with a loss of β–lactam resistance, although this reconstruction was ambiguous. Similarly, of the eleven recombinations that spanned at least part of the *pbp1a* gene, three caused no change in serotype. One of these ‘silent’ events was temporally associated with other recombinations affecting *pbp2x* and *pbp2b*, leading to the serotype 23F SC9 isolate R34-3013 developing β–lactam resistance [[1](#_ENREF_1)].

**Supplementary References**

1. Croucher NJ, Finkelstein JA, Pelton SI, Mitchell PK, Lee GM, et al. (2013) Population genomics of post-vaccine changes in pneumococcal epidemiology. Nature genetics 45: 656-663.
